# A pooled shRNA screen for regulators of primary mammary stem and progenitor cells identifies roles for *Asap1* and *Prox1*

**DOI:** 10.1186/s12885-015-1187-z

**Published:** 2015-04-03

**Authors:** Julie M Sheridan, Matthew E Ritchie, Sarah A Best, Kun Jiang, Tamara J Beck, François Vaillant, Kevin Liu, Ross A Dickins, Gordon K Smyth, Geoffrey J Lindeman, Jane E Visvader

**Affiliations:** 1 ACRF Stem Cells and Cancer Division, The Walter and Eliza Hall Institute of Medical Research, 1G Royal Parade, Parkville, VIC 3052 Australia; 2 Molecular Genetics of Cancer Division, The Walter and Eliza Hall Institute of Medical Research, 1G Royal Parade, Parkville, VIC 3052 Australia; 3 Bioinformatics Division, The Walter and Eliza Hall Institute of Medical Research, 1G Royal Parade, Parkville, VIC 3052 Australia; 4 Molecular Medicine Division, The Walter and Eliza Hall Institute of Medical Research, 1G Royal Parade, Parkville, VIC 3052 Australia; 5 Department of Medical Biology, The University of Melbourne, Parkville, VIC 3010 Australia; 6 School of Mathematics and Statistics, The University of Melbourne, Parkville, VIC 3010 Australia; 7 Department of Medicine, The University of Melbourne, Parkville, VIC 3010 Australia; 8 Department of Medical Oncology, The Royal Melbourne Hospital, Grattan Street, Parkville, VIC 3050 Australia

**Keywords:** Mammary stem cells, Mammary progenitor cells, Transcription factors, Mammosphere, shRNA screen, *Asap1*, *Prox1*

## Abstract

**Background:**

The molecular regulators that orchestrate stem cell renewal, proliferation and differentiation along the mammary epithelial hierarchy remain poorly understood. Here we have performed a large-scale pooled RNAi screen in primary mouse mammary stem cell (MaSC)-enriched basal cells using 1295 shRNAs against genes principally involved in transcriptional regulation.

**Methods:**

MaSC-enriched basal cells transduced with lentivirus pools carrying shRNAs were maintained as non-adherent mammospheres, a system known to support stem and progenitor cells. Integrated shRNAs that altered culture kinetics were identified by next generation sequencing as relative frequency changes over time. RNA-seq-based expression profiling coupled with *in vitro* progenitor and *in vivo* transplantation assays was used to confirm a role for candidate genes in mammary stem and/or progenitor cells.

**Results:**

Utilizing a mammosphere-based assay, the screen identified several candidate regulators. Although some genes had been previously implicated in mammary gland development, the vast majority of genes uncovered have no known function within the mammary gland. RNA-seq analysis of freshly purified primary mammary epithelial populations and short-term cultured mammospheres was used to confirm the expression of candidate regulators. Two genes, *Asap1* and *Prox1*, respectively implicated in breast cancer metastasis and progenitor cell function in other systems, were selected for further analysis as their roles in the normal mammary gland were unknown. Both *Prox1* and *Asap1* were shown to act as negative regulators of progenitor activity *in vitro*, and *Asap1* knock-down led to a marked increase in repopulating activity *in vivo*, implying a role in stem cell activity.

**Conclusions:**

This study has revealed a number of novel genes that influence the activity or survival of mammary stem and/or progenitor cells. Amongst these, we demonstrate that *Prox1* and *Asap1* behave as negative regulators of mammary stem/progenitor function. Both of these genes have also been implicated in oncogenesis. Our findings provide proof of principle for the use of short-term cultured primary MaSC/basal cells in functional RNAi screens.

**Electronic supplementary material:**

The online version of this article (doi:10.1186/s12885-015-1187-z) contains supplementary material, which is available to authorized users.

## Background

The mammary epithelial tree is a bilayered, branched structure composed of an outer myoepithelial (basal) layer and an inner luminal layer. The full differentiative potential of the mammary gland is manifest in response to pregnancy hormones, when a subset of luminal cells gives rise to alveolar cells that produce milk, which is then extruded through the lumena during lactation. The prospective isolation of mammary stem cells (MaSCs) that are able to give rise to an entire mammary tree upon transplantation at the single cell level [1,2] and the phenotypic identification of several mammary epithelial progenitor cell (MaPC) populations [3-6] have enhanced our current understanding of the differentiation hierarchy. More recently, *in vivo* genetic tracing experiments have demonstrated the existence of bipotent MaSCs [7,8] and long-lived progenitors [7,9,10] that contribute to morphogenesis in puberty and pregnancy, and ductal maintenance in the adult gland. However, the molecular processes underpinning the functions of stem and progenitor cells remain poorly understood.

Genetic manipulation and pathway interference have been successfully used at the level of single genes to determine the role of regulators of mammary gland morphogenesis (reviewed in [11]). RNAi screening has provided novel molecular insights in different cellular systems but large-scale or genome-wide screens have not yet been performed in the context of primary mammary epithelial cells. Rather, such screening strategies have been restricted to mammary epithelial and breast cancer cell lines, which offer the advantages of being readily available and amenable to genetic manipulation [12-15]. In other organs, primary cells have been used in RNAi screens to study tissue stem and progenitor cell behavior in more complex and physiological contexts [16-19]. To explore novel molecular regulators of MaSCs and MaPCs, we have utilized a targeted shRNA library to interrogate freshly isolated MaSC-enriched cells *ex vivo*. This study supports the use of large shRNA libraries to identify novel regulators of mammary epithelial function using a non-adherent mammosphere-based assay and has revealed several novel regulators of MaSC/basal cell function.

## Results

### A pooled shRNA screen for the identification of regulators of mammary stem/progenitor cells using primary cells

**Figure 1:**
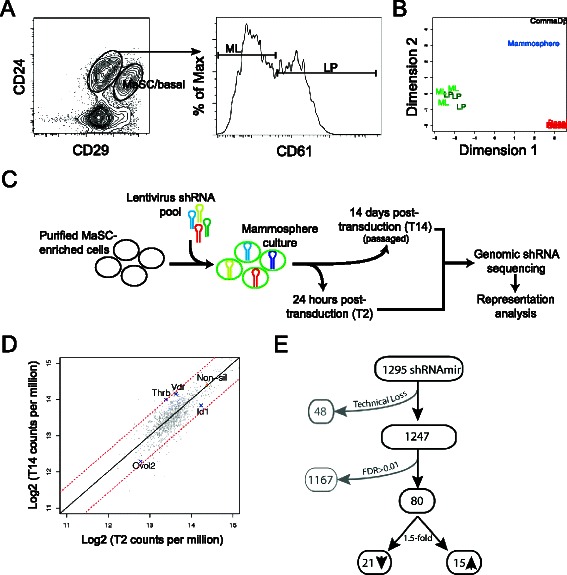
**Differential RNA-seq expression analysis of sorted subpopulations and mammospheres and pooled shRNA screening strategy. (A)** Flow cytometric profiles of Lin^−^ mammary cell populations showing representative sort gates for CD29^hi^CD24^+^ MaSC-enriched basal cells (MaSC/basal), CD29^lo^CD24^+^CD61^+^ luminal progenitor-enriched cells (LP) and CD29^lo^CD24^+^CD61^−^ mature luminal cell-enriched cells (ML). **(B)** Multidimensional scaling plot of expression data generated by RNA-Seq of populations including MaSC/basal (Basal), LP and ML populations, MaSC/basal-derived mammospheres (Mammosphere) and Comma-Dβ cells (CommaDβ). **(C)** Schematic outline of the screening strategy. **(D)** Plot showing the relative frequency of shRNA-carrying cells at T2 and T14 as determined by next-generation sequencing, including a non-silencing control hairpin (non-sil). Dashed lines, 1.5-fold change. **(E)** Numerical summary of shRNA performance in the screen.

To identify novel regulators of mammary epithelial stem and progenitor cells, we utilized a GIPZ mouse transcription factor gene shRNA library to perform a screen largely based on proliferation/survival potential using primary mammary epithelial cells. We selected the non-adherent mammosphere assay, which is principally a progenitor assay but is also permissible for the maintenance and differentiation of stem cells [20-22] upon short-term culture. Freshly isolated cells in the CD29^hi^CD24^+^ subset (Figure 1A) enriched in transplantable MaSCs, myoepithelial cells and other basal intermediates (MaSC/basal) [1] were first tested in the mammosphere system to study their clonogenic properties *ex vivo*. Following culture in mammosphere medium, MaSC/basal cells retained the ability to generate colonies in both 2D assays on irradiated NIH/3T3 feeder layer and 3D Matrigel assays designed to detect MaPC activity (data not shown and Additional file 1: Figure S1A). Importantly, upon transplantation, the ability of mammosphere cells to repopulate a mammary fat pad was maintained during culture at a frequency of 1 in 298 mammosphere cells (Additional file 1: Figure S1B and C).

The suitability of the mammosphere system for a large scale shRNA library screen was next investigated by RNA-seq analysis of freshly sorted MaSC/basal cells; luminal MaPCs (CD29^lo^CD24^+^CD61^+^; LP); a mature luminal cell-enriched (CD29^lo^CD24^+^CD61^−^; ML) population; mammosphere-derived cells generated from MaSC/basal cells harvested after 7 days in non-adherent culture; and the Comma Dβ cell line, which comprises bipotent cells capable of mammary reconstitution [23] (Figure 1B). Comparative analysis revealed that mammosphere cells had an expression profile intermediary to basal and luminal cell populations indicating that some luminal lineage gene expression had been initiated during culture (Figure 1B). The propensity of MaSC/basal-derived mammosphere culture to support commitment to the luminal lineage was demonstrated by the appearance of colonies with an acinar morphology identical to those derived from luminal MaPCs in Matrigel cultures (Additional file 1: Figure S1A and data not shown). Notably, global gene expression in the primary mammary epithelial subsets was more similar to mammosphere cells than to the Comma Dβ cell line, suggesting that primary cell-initiated mammospheres represent a more physiological screening platform than established cell lines (Figure 1B). Comparison of RNA-seq expression profiles with previously reported microarray profiles (Illumina MouseWG-6 v2.0 BeadChip platform [24]) revealed a strong correlation between the two technologies, however, RNA-seq demonstrated a greater dynamic expression range and an increased number of differentially expressed transcripts (Additional file 2: Figure S2).

To identify genes that influenced the proliferation or survival of freshly sorted MaSC/basal cells in mammosphere culture, we screened a customized mouse lentiviral library consisting of 1,295 shRNAmirs mostly targeting transcription factors and represented in 15 pools (Figure 1C). Two rounds of infection of 2 × 10^6^ cells resulted in a transduction frequency of ~40% (data not shown). Transduced cells were harvested at 24 h or 12 days following the second transduction, and representation of integrated shRNAs was assessed using PCR from genomic DNA and next generation sequencing. Adapter and short index sequences in the PCR primers permitted multiplexing of samples (Figure 1C). Following next generation sequencing, shRNA read counts within each indexed sample were determined and changes in shRNA frequency over time were identified in pre- and post-culture samples (Figure 1C, D and Additional file 3: Table S1). With 0.85% of freshly isolated cells expected to give rise to primary mammospheres and assuming a 40% infection rate with a pool of 88 shRNAs, the number of interrogated mammospheres harboring a particular shRNA would be expected to be above 77 in each replicate experiment. Of note, three to five biological replicates were prepared for each of the 15 pools yielding a total of 102 samples.

From a total of 1,295 shRNAs analyzed in the screen, sequence read data was obtained for 1,247 shRNAs (Figure 1E and Additional file 3: Table S1). Eighty shRNAs targeting 73 genes significantly altered sphere growth (FDR < 0.01), with 15 shRNAs conferring a >1.5-fold growth advantage and a 21 shRNAs showing a >1.5-fold reduced prevalence (Figure 1D, E and Table 1). Among deleterious shRNAs were those targeting essential genes such as the TATA binding protein *(Tbp),* which is required for transcription (Table 1). Notably, several known regulators of mammary gland morphogenesis and/or epithelial proliferation, such as *Ovol2* [25] and *Id1* [26,27], were found to be significantly depleted (Figure 1D and Table 1). Moreover, basally-expressed transcription factors (*Tcf4* and *Lef1*) that are implicated in mammary stem cell renewal through the Wnt pathway were depleted in the functional screen [28]. Although *Snai2* has been shown to be a positive regulator of MaSCs, it was not detected in our screen, likely reflecting inefficient knock-down by the two targeting shRNA hairpins present in the library. Conversely, we observed enrichment of shRNAs targeting genes previously associated with mammary hyperplasia in knockout mouse models including *Thrb* [29] and *Vdr* [30] (Figure 1D and Table 1). Several genes with reported roles in stem cell renewal and differentiation in other organ systems were also revealed by the mammosphere screen, including *Prox1* [31,32] and *MafB* [33]*.*

**Table 1:** **Table of shRNA clones eliciting frequency changes with a FDR ≤ 0.01**

Vendor clone ID	Gene	logFC	logCPM	PValue	FDR
V2LMM_6871	MYOG	−2.5393	9.6584	5.24E-39	4.61E-37
V2LMM_68084	ASAP1	1.2699	13.5019	7.53E-29	6.62E-27
V2LMM_63192	LASS6	−1.5047	12.8189	3.47E-23	3.06E-21
V2LMM_64405	NR0B1	−1.2213	12.6156	3.74E-14	3.29E-12
V2LMM_189334	SOX21	0.7841	14.1110	7.57E-11	6.66E-09
V2LMM_82812	Id1	−0.4067	14.0413	1.58E-10	9.96E-09
V2LMM_73029	Myst1	−0.3976	13.7299	4.37E-10	1.38E-08
V2LMM_62263	TBP	−0.9262	6.8162	5.90E-10	2.60E-08
V2LMM_25195	TCEA2	−0.9252	13.0207	6.44E-10	2.69E-08
V2LMM_13506	NKX3-1	−0.9163	13.0783	9.16E-10	2.69E-08
V2LMM_54422	POU1F1	−0.9631	12.8253	1.91E-09	8.39E-08
V2LMM_9553	FOXN1	−1.0459	12.0477	8.86E-09	3.90E-07
V2LMM_259235	FOXC2	0.6794	11.6227	3.67E-08	1.61E-06
V2LMM_53719	TEF	−0.7084	12.9529	3.54E-08	2.24E-06
V2LMM_27467	NFE2L1	−0.7027	12.1790	5.10E-08	2.24E-06
V2LMM_937	MNT	0.8370	13.6556	1.60E-07	4.71E-06
V2LMM_20404	MAFG	0.7503	14.4488	4.96E-07	1.09E-05
V2LMM_218286	SOX9	0.5992	14.6443	5.34E-07	1.57E-05
V2LMM_29411	Ezh2	0.3074	13.6843	1.37E-06	2.89E-05
V2LMM_64907	NR1H3	−0.6947	13.0997	3.26E-06	5.74E-05
V2LMM_226932	NOTCH2	−0.7216	12.6071	7.35E-07	6.47E-05
V2LMM_22166	NR1H4	0.6672	14.0499	7.62E-06	1.12E-04
V2LMM_70598	Klf4	−0.2842	13.7153	7.81E-06	1.23E-04
V2LMM_29885	Lmo2	−0.2790	13.3138	1.25E-05	1.57E-04
V2LMM_85045	ZFP449	−0.5770	13.6233	6.17E-06	1.81E-04
V2LMM_27178	TRPS1	−0.7064	12.9042	9.70E-06	2.13E-04
V2LMM_7277	NRARP	0.5023	13.3971	8.02E-06	2.35E-04
V2LMM_71087	LEF1	−0.6382	13.1210	1.87E-05	2.35E-04
V2LMM_37331	OVOL2	−0.4958	12.5577	1.11E-05	2.45E-04
V2LMM_82311	FOXJ1	−0.4883	13.7075	1.41E-05	2.48E-04
V2LMM_30422	Ezh2	0.2667	13.9399	2.73E-05	2.87E-04
V2LMM_86479	HLF	0.6524	13.6835	6.89E-06	3.03E-04
V2LMM_73715	KLF5	−0.6192	12.8163	3.27E-05	3.60E-04
V2LMM_77459	Tcf4	−0.2612	13.7487	4.01E-05	3.61E-04
V2LMM_87315	ANKRD46	0.6747	13.9989	2.27E-05	3.99E-04
V2LMM_249987	ANKRD33	0.5109	14.5653	1.82E-05	4.00E-04
V2LMM_34449	THRB	0.6078	13.7210	4.51E-05	4.32E-04
V2LMM_34394	MRG2	0.6048	13.8720	4.91E-05	4.32E-04
V2LMM_71843	TCF19	−0.5495	13.0225	6.03E-06	5.31E-04
V2LMM_162758	ETS1	0.5252	13.3887	1.45E-05	6.38E-04
V2LMM_103225	Rbbp8	0.2503	13.5222	8.51E-05	6.70E-04
V2LMM_3550	TCF21	0.4517	13.0423	5.97E-05	8.76E-04
V2LMM_61869	TBP	−0.6028	13.2274	3.29E-05	9.64E-04
V2LMM_110117	NFATC3	−0.7358	13.2152	4.56E-05	1.34E-03
V2LMM_70871	NR1I3	−0.6202	13.2914	9.95E-05	1.36E-03
V2LMM_196851	GATA6	0.6165	13.4939	1.08E-04	1.36E-03
V2LMM_86811	Klf8	−0.2331	14.0270	2.43E-04	1.61E-03
V2LMM_14204	Cbx4	0.2323	14.0165	2.55E-04	1.61E-03
V2LMM_103224	Rbbp8	−0.2249	13.7322	4.06E-04	2.33E-03
V2LMM_2021	MTA3	0.5498	13.3661	2.69E-05	2.37E-03
V2LMM_18790	CLOCK	0.6957	14.2333	1.13E-04	2.49E-03
V2LMM_8902	2410018C20RIK	−0.4430	13.8888	1.46E-04	2.57E-03
V2LMM_7952	NR2C1	0.6810	13.8576	1.57E-04	2.76E-03
V2LMM_65378	E2F6	0.4731	12.6660	9.67E-05	2.84E-03
V2LMM_14131	Id3	0.2176	14.1862	6.16E-04	3.23E-03
V2LMM_4616	Cbx4	0.2158	14.2122	6.73E-04	3.26E-03
V2LMM_81587	Klf9	−0.2144	13.9803	7.36E-04	3.31E-03
V2LMM_50581	NR2F2	−0.5745	12.8242	3.14E-04	3.45E-03
V2LMM_71013	HNRPAB	−0.5309	13.3310	8.03E-05	3.53E-03
V2LMM_71313	CML3	−0.6586	13.3786	2.59E-04	3.80E-03
V2LMM_46526	PROX1	0.6463	13.6892	3.35E-04	4.21E-03
V2LMM_63443	MAFB	−0.4007	13.4709	3.65E-04	4.59E-03
V2LMM_75410	ASB4	−0.3952	13.3161	4.41E-04	4.85E-03
V2LMM_87318	ANKRD46	0.3883	13.7227	5.46E-04	5.11E-03
V2LMM_88650	6430502M16RIK	−0.3867	13.5604	5.81E-04	5.11E-03
V2LMM_194038	HOXD8	0.4901	13.8093	1.84E-04	5.39E-03
V2LMM_71230	NR1I3	−0.6275	12.9976	5.00E-04	5.50E-03
V2LMM_79666	PAX8	−0.5296	13.7022	2.58E-04	5.67E-03
V2LMM_64571	GCDH	0.4455	13.2693	2.62E-04	5.76E-03
V2LMM_50262	VDR	0.5204	13.8960	3.27E-04	5.76E-03
V2LMM_66887	LASS4	−0.4153	13.1732	3.99E-04	5.85E-03
V2LMM_71060	GTF2H4	−0.4379	13.3758	3.04E-04	6.58E-03
V2LMM_5760	MYC	0.4271	13.4915	4.15E-04	6.58E-03
V2LMM_6641	PYCARD	0.4248	13.2027	4.49E-04	6.58E-03
V2LMM_29358	TBX20	−0.5067	13.3472	4.76E-04	6.98E-03
V2LMM_212338	PPP1R16B	0.3734	13.6841	8.86E-04	7.09E-03
V2LMM_68781	OTX1	−0.6004	13.2971	8.58E-04	8.39E-03
V2LMM_71737	HOXA5	0.4088	13.7302	7.23E-04	9.08E-03
V2LMM_67978	LASS4	0.3995	12.8183	9.89E-04	9.91E-03
V2LMM_62940	IRX6	−0.3977	13.5485	1.01E-03	9.91E-03

**Figure 2:**
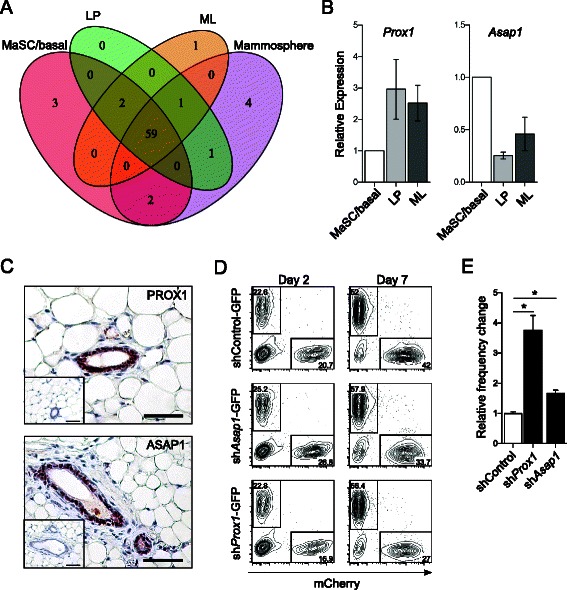
**Selection of candidate genes for further analysis. (A)** Venn diagram summarizing expression of genes, as determined by RNA-Seq, targeted by shRNAs with FDR < 0.01 in freshly isolated MaSC/basal, LP and ML populations and MaSC/basal-derived mammosphere (Mammosphere) populations. **(B)** qRT-PCR profiling of two candidate regulators, *Prox1* and *Asap1*, in primary epithelial subsets (n = 3; mean ± S.E.M). **(C)** Immunohistochemistry showing PROX1 and ASAP1 protein expression in mammary epithelial cells of 8-week-old virgin mice. **(D)** Representative FCM plots showing the relative abundance of MaSC/basal cells transduced with sh*Control*-GFP, sh*Prox1*-GFP or sh*Asap1*-GFP retrovirus and competitor cells transduced with control mCherry-expressing retrovirus at Day 2 and Day 7 in i3T3 cultures. **(E)** Histogram showing the change in the ratio of shRNA-GFP^+^: mCherry^+^ cells for each shRNA between 2 and 7 days of co-culture. Data from 3 independent experiments displayed as mean ± S.E.M. Statistical significance was calculated relative to sh*Control:* sh*Prox1*, p ≤ 0.035; sh*Asap1*, p ≤ 0.013.

To eliminate potential false-positives, RNA-seq was used to confirm the expression of candidate regulators in freshly isolated MEC subpopulations. Candidate genes with average counts per million (CPM) >0.5 were deemed to be expressed and considered potential regulators. Of the 73 genes targeted by shRNAs, 68 were expressed in one or more of the epithelial populations with 63 (93%) also expressed by mammospheres (Figure 2A). Additionally, a further four genes (6%) were expressed in mammospheres but not primary cells, indicating potential upregulation of these genes during mammosphere culture or selection of a rare cell type through culture (Figure 2A). Seven (10%) shRNAs with a FDR < 0.01 targeted genes that were not expressed at an appreciable level in any population, suggesting shRNA off-target effects (data not shown).

### *In vitro* validation of two candidate regulators, *Asap1* and *Prox1*

Two candidates, *Asap1* (ARF-GAP protein with SH3 domains, ankyrin repeats and plekstrin homology domain) and *Prox1* (Prospero homeobox 1) were chosen for further study. Hairpins against either of these genes were enriched during the screen, indicating that their knock-down promoted the proliferation/survival of basal epithelial cells. *Asap1* is a multi-domain member of the ARF-GAP protein family and has roles in metastasis in several systems including breast cancer cell lines, in which it has been implicated in invasion and metastatic potential [34]. However, a role for *Asap1* in normal developmental processes has not yet been described. *Prox1* is a homeobox transcription factor that exerts multiple roles in different organs including lineage specification [31,35] and maintenance of lineage identity, but its role in the mammary gland also remains unknown.

The screen demonstrated that cells carrying sh*Asap1* increased in frequency nearly 2.5-fold (FDR, 7.1 × 10^−27^) whereas sh*Prox1*-carrying cells increased more than 1.6-fold (FDR, 4.2 × 10^−3^; Table 1). Expression profiling confirmed that *Asap1* and *Prox1* were expressed in all mammary epithelial subpopulations but showed differential expression between the MaSC/basal and luminal subpopulations (Figure 2B and C). To validate shRNA representation differences observed in the screen, individual shRNAs were first evaluated in a competitive cell assay for cell growth. Over the course of 14 days in culture, the relative abundance of sorted MaSC/basal cells transduced with virus-encoded shRNA-GFP versus a reference population of MaSC/basal cells transduced with a virus-encoded mCherry fluorescent protein was measured by flow cytometry (Figure 2D). Changes in the ratio of shRNA-GFP^+^: mCherry^+^ cells revealed the effect of shRNAs on cell ‘fitness’ (Figure 2D and E). To avoid potential silencing of the CMV promoter that drives shRNA and GFP expression in the pGIPZ lentiviral vector, shRNAs were re-cloned into the retroviral LMS vector, which remains active in mammary epithelial cells throughout culture and is permissive for the maintenance of stem and progenitor cells [36]. Sorted MaSC/basal cells were plated on an irradiated NIH/3T3 (i3T3) monolayer to support their growth and then transduced. Consistent with our screen results, cells carrying sh*Asap1* or sh*Prox1* were enriched during co-culture and both shRNAs stimulated colony growth at day 7 and 14 after plating (Figure 2C, D and data not shown). The relative numbers of shRNA-GFP^+^ cells for sh*Prox1* were expanded by approximately 4-fold following a short culture period of 5 days (p = 0.028) (Figure 2D and E), while shAsap1 conferred a more modest advantage of 1.5-fold (p = 0.011) (Figure 2D and E). As expected, a non-silencing control shRNA conferred no advantage on transduced cells (Figure 2D and E).

### *Prox1* inhibits the clonogenic potential of mammary epithelial cells

**Figure 3:**
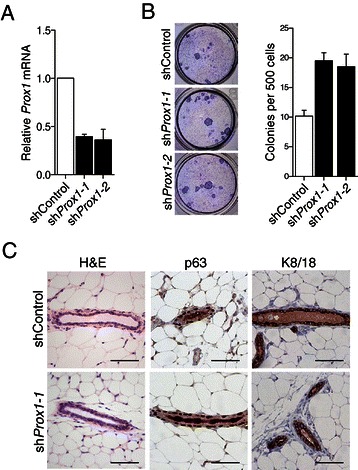
***Prox1*is a negative regulator of mammary epithelial progenitor cells*in vitro.*(A)** qRT-PCR detection of *Prox1* transcript abundance in MaSC/basal cells following transduction with retroviruses expressing sh*Control* or shRNAs targeting *Prox1*. Data are shown as mean ± S.E.M. *Prox1* expression normalized to *Ywhaz1* expression relative to sh*Control* (n = 2). **(B)** Transduced cells (500) were plated on an i3T3 feeder layer and cultured for 6 days to allow the formation of colonies. Left panel: representative images of observed colonies. Right panel: histogram showing the number of colonies derived from cells transduced with sh*Control*- and sh*Prox1*-retroviruses. Data are shown as mean ± S.D for three independent experiments. **(C)** Haematoxylin and eosin staining and anti-P63 (myoepithelial) and anti-K8/K18 (luminal) immunohistochemical staining of outgrowths derived from cells transduced with a control versus sh*Prox1* retrovirus. Scale bars, 50 μm.

Two shRNAs against *Prox1* (sh*Prox1-1* and sh*Prox1-2*) that reduced *Prox1* expression to below 40% of wild-type levels were selected for further clonogenic assays on i3T3 feeder layers (Figure 3A). Initially, an established regulator of mammary progenitor activity, *Snai2* [37] was tested in this system using two shRNAs (sh*Snai2*-1 and sh*Snai2*-2) (Additional file 4: Figure S3A and B). An 80% reduction in clonogenicity was observed with these hairpins, supporting the efficacy of knockdown and clonogenic readout in this system (Additional file 4: Figure S3C). Cells carrying either *Prox1* shRNA demonstrated a ~ two-fold higher clonogenicity than those carrying a non-silencing control shRNA (Figure 3B). Transplantation of MaSC/basal cells transduced with sh*Prox1*-expressing retroviruses yielded outgrowths with normal morphology and did not reveal any difference in repopulating frequency compared to control cells (Figure 3C and data not shown). These findings suggest that *Prox1* levels are less critical for the activity of MaSCs than MaPCs, although the effect of reducing *Prox1* expression to even lower levels is yet to be determined.

### *Asap1* suppresses mammary stem and progenitor cell numbers or activity

**Figure 4:**
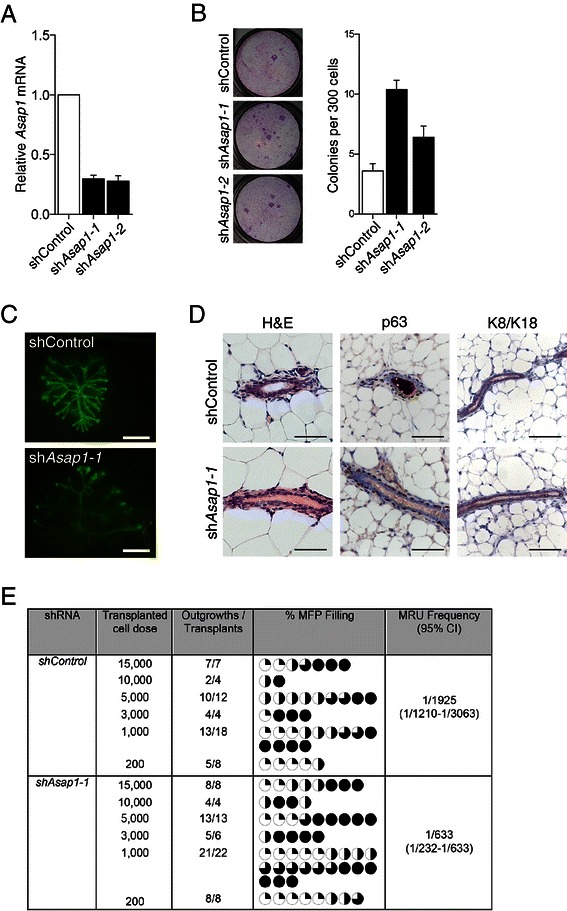
***Asap1*negatively regulates mammary stem and progenitor cells. (A)** Quantitative RT-PCR detection of *Asap1* transcript abundance in MaSC/basal cells following transduction with retroviruses expressing sh*Control* or shRNAs targeting sh*Asap1.* Data are shown as mean ± S.E.M. (n = 3). *Prox1* expression is normalized to *Ywhaz1* expression relative to sh*Control*. **(B)** Left panel: transduced cells were plated on an i3T3 feeder layer and cultured for 6 days to allow the formation of colonies. Right panel: histogram showing the number of colonies derived from sh*Control*- and sh*Asap1*-transduced cells. Data are shown as mean ± S.D for 3 independent experiments. **(C)** Representative whole-mount images of GFP^+^ outgrowths derived from transplantation of sh*Control*- or sh*Asap1-*retrovirally transduced MaSC/basal cells. Scale bar, 2 mm. **(D)** Morphological analysis of outgrowths. Haematoxylin and eosin staining and immunohistochemical staining for P63 and K8/K18 of outgrowths following transplantation. Scale bars, 50 μm. **(E)** Table of limiting dilution analysis of transplantation frequencies of MaSC/basal cells transduced with sh*Control* or sh*Asap1* retroviruses. The number of transplants and resulting outgrowths is shown as well as the extent of fat pad filling by individual outgrowths.

Two independent shRNAs that reduced *Asap1* expression to approximately 25% of wild-type levels (Figure 4A) were used to confirm a role for *Asap1* in normal primary mammary epithelial cells. Transduction of MaSC/basal cells with either sh*Asap1-1* or sh*Asap1-2* retrovirus resulted in higher progenitor numbers compared to control cells in 2D clonogenic assays (Figure 4B), confirming a role for *Asap1* in progenitor cells. Transplantation of sh*Asap1*-transduced cells into clear fat pads revealed a greater than 3-fold higher repopulation frequency compared to sh*Control* virus-infected cells (Figure 4C). Branched GFP^+^ outgrowths were morphologically similar to those transduced with sh*Control* retrovirus and exhibited a similar degree of fat-pad filling (Figure 4C). The typical architecture of these outgrowths was confirmed by immunohistochemical staining, with an outer layer of myoepithelial cells expressing p63 and SMA, and a luminal cell layer expressing Cytokeratin 8/18 and E-Cadherin (Figure 4D and data not shown). Moreover, outgrowths derived from *Asap1* knock-down cells were capable of full differentiation to milk-producing alveoli when recipients were subject to pregnancy (data not shown).

## Discussion

In this study, we have developed a protocol to identify novel regulators of mammary stem/progenitor cells using freshly isolated MaSC-enriched cells for a functional RNAi screen based on pooled shRNA libraries. Based on three independent biological screens, we identified shRNAs targeting 73 genes as potential modulators of stem/progenitor cell behavior with more than half of those targeting novel genes that have not been previously implicated in mammary gland development. Although the changes were modest, they were highly reproducible. Notably, the strategy also identified a number of known regulators of stem and progenitor cells, thereby validating the screening strategy. The mammosphere assay primarily reads out progenitor activity, given that the transplantation frequency of mammosphere cultures is approx. 1 in 300 (read-out for stem cells) whereas the colony-forming potential of these cells is around 1 in 20 (read-out for stem/progenitor cells). The system established here should be immediately applicable to future sgRNA/CRISPR libraries using pooled screens [38].

Potential limitations associated with this and other shRNA-based functional screens, include poor coverage of genes by multiple shRNAs (in this case a mean shRNA per gene of 2; mode, 1), and incomplete knock-down of gene expression. The observed modest fold changes in part reflect the use of primary cells in a short-term mammosphere assay, which is necessary to obviate any changes associated with prolonged culture of epithelial cells, resulting in smaller amounts of material post-culture relative to that obtained from the use of established cell lines. It is noteworthy that the fold-changes observed here are comparable to those observed in another *in vitro* shRNA screen at early time-points [17].

Further exploration of two genes with verified expression in the mammary gland, *Asap1* and *Prox1,* revealed roles in regulating mammary basal progenitor activity. Retrovirus-mediated knockdown of either gene augmented progenitor cell numbers in colony forming assays *in vitro*. Moreover, knockdown of *Asap1* expression led to a significant increase in the repopulating frequency, suggesting that *Asap1* either negatively regulates MaSC numbers or their activity. Conversely, knockdown of *Prox1* did not affect mammary repopulating potential, either suggesting that *Prox1* does not compromise MaSC function or that complete knock-down of this gene is required for an overt phenotype. In other organs, there is evidence that *Prox1* regulates stem and/or progenitor cell activity in a context-dependent fashion (reviewed in [39]). Interestingly, both genes have been postulated to contribute to oncogenesis when overexpressed. ASAP1 has been shown to be necessary for the *in vitro* invasive potential and *in vivo* metastatic potential of specific breast cancer cell lines including MDA-MB-231 cells (Onodera et al., 2005), while increased *Prox1* expression promotes the transition of intestinal adenomas to high-grade dysplasia or carcinoma *in situ* [40]. Additional experiments using inducible gene knock-out strategies or CRISPR/Cas9 technology to further reduce or ablate ASAP1 or PROX1 protein levels will be required to clarify the specific effects of these genes on distinct mammary cell populations during normal development and to elucidate their roles in breast oncogenesis.

## Methods

### Mammary cell preparation and cell culture

The preparation of mammary epithelial cell suspensions from 8–10 week-old FVB/N female mice and flow cytometric purification has been described [1]. Unless otherwise stated, all chemicals and media components were purchased from Life Technologies (Carlsbad, CA, USA) or Sigma (St Louis, MO, USA). For mammosphere culture, cells were plated in ultra-low adherence plates (Corning) in mammosphere medium (DMEM/F12 + Glutamax, 1% penicillin/streptomycin, 10 ng/ml EGF, 10 ng bFGF, 5 μg/ml insulin, 0.5 μg/ml hydrocortisone, B27 supplement) at 100,000 cells/ml and maintained as suspension cultures. Medium was exchanged every 3–4 days and spheres were passaged using trypsin-EDTA and gentle trituration every 7 days before replating at a density of not greater than 50,000 cells/ml. For irradiated NIH/3T3 (i3T3) co-culture, cells were counted manually and plated in tissue culture plates with i3T3 fibroblasts (5,000 Rads) in mammary growth medium (DMEM/F12 with glutamax, 1% penicillin/streptomycin, 10 ng/ml EGF, 5 μg/ml insulin, 0.5 μg/ml hydrocortisone, 20 ng/ml cholera toxin) with 5% FCS. After overnight incubation at 37°C in 5% O_2_/5% CO_2_, the cultures were changed to the same medium containing 1% FCS and incubated for a further 5 days. Colonies were harvested with trypsin/EDTA for sorting or stained with Giemsa for imaging and colony enumeration. For 3D colony assays, transduced cells were mixed with Matrigel (BD Biosciences, San Jose, CA) and cultured as described [1,11]. Cultures were imaged before fixation with 4% paraformaldehyde and embedded in paraffin. CommaDβ cells were maintained as previously described [23].

### Transplantation and mammary gland outgrowth analysis

GFP-expressing transduced cells sorted by flow cytometry were manually counted and transplanted at limiting dilution as described [1], in the presence of 25% growth factor-reduced Matrigel. GFP^+^ outgrowths were visualized using a dissecting microscope (Leica Microsystems Gmbh, Wetzlar, Germany) and histology performed as described [3]. Mammary fat pad filling was quantitated comparing total fat pad area and outgrowth area using Image J software. All animal experiments conformed to regulatory standards and were approved by the Walter and Eliza Hall Institute (WEHI) Animal Ethics Committee.

### Lentivirus production and transduction

A library of GIPZ plasmids expressing shRNAs was expanded individually in bacteria, then clones were pooled and plasmids purified yielding 15 pools of 90 shRNAs (Open Biosystems Transcription Factors Gene Family Library cat#RMM4950) and one pool of 63 shRNAs (Open Biosystems, custom order WEHI_73597). Lentivirus production was initiated by calcium phosphate transfection of 293T cells with pGIPZ shRNA-containing vectors and pMD2.G and psPAX2 (Addgene plasmids 12259 and 12260). Viral supernatants were collected at 26 and 44 hr post-transfection and concentrated via ultracentrifugation as per manufacturer’s protocol. Pellets were resuspended in mammary growth medium with 5% FCS, centrifuged at maximum speed for 5 minutes at room temperature to remove most serum proteins. Supernatant containing 100× concentrated virus stored at −80°C. The titre of each frozen virus stock was assessed biologically. Briefly, the day prior to transduction, 50,000 293T cells were seeded into 12-well plates in 293T medium (DMEM with 10% FCS and 1% penicillin/streptomycin). On the day of transduction, cells in three wells were counted to determine the number of cells present at transduction. Dilutions of virus made in 293T medium containing 5 μg/ml polybrene were used to replace the medium on remaining wells so that a series of wells were exposed to decreasing quantities of virus. Following transduction for 16–24 hr, the medium was changed and 48 hr later, cells were analyzed for GFP expression by flow cytometry. The number of cells at transduction and the amount of virus added to a well, where the percentage of GFP^+^ cells was between 1 and 20%, was used to calculate the transducing units (TU) per ml using the following formula: [Number of cells at transduction × (% GFP^+^ cells/100)]/volume of virus (ml). Typical TU of 1 × 10^8^/ml were achieved. For mammosphere transduction, 2 × 10^6^ purified cells were plated in mammosphere medium containing 5 μg/ml polybrene and transduced at 0 and 16 hr with 4 × 10^6^ TU. Medium was exchanged after 24 hr and a sample of the culture was taken for analysis of baseline shRNA frequency (time-point T2). Following 14 days in culture, the remaining cells were harvested (T14).

### Retrovirus cloning, production and transduction

The LMS vector into which the non-silencing, and targeting shRNAs were cloned has been described [41] and the LMS-sh*Control*, containing a non-specific shRNA sequence, was obtained from Open Biosystems. Other shRNA templates were designed as described and PCR amplified from DNA oligonucleotides using the forward primer 5’-CAGAAGGCTCGAGAAGGTATATTGCTGTTGACAGTGAGCG-3’ and the reverse primer 5’-CTAAAGTAGCCCCTTGAATTCCGAGGCAGTAGGCA-3’ [42]. Alternatively, shRNAs were purchased from OpenBiosystems as pGIPZ clones. In both cases, 137 bp shRNA products were isolated using EcoRI and XhoI digestion and subcloned into the LMS vector. Mature anti-sense sequences were as follows: *shAsap1-1* (Open Biosystems clone V3LMM_492766), 5’-TTCGTCGTCATTATCTGCCTGG-3’; *shAsap1-2*, 5’-ATATTATATAAGTCAGCAGCCA-3’; *shProx1-1* (Open biosystems clone V3LMM_506363), 5’-TTCTCTGTAACTTTCTCGG-3’; *shProx1-2* (Open Biosystems clone V3LMM_506365), 5’-TTCACTCCAATGTCAACCC-3’; *shSnai2-1* described previously [43]; *shSnai2-2* 5’-CGCAGACCCACTCTGATGTAA-3’. For production of retroviruses, Phoenix cells (10 cm plates) were transfected with 5 μg of vector using calcium phosphate precipitation and the virus supernatant was collected and filtered through a 0.45 μm filter at 48 and 72 hr. For transduction, cells on feeder layers in 6 well-plates were spin infected (2,500 rpm, 32°C, 1 hr) with 2 ml of viral supernatant per well containing 5 μg/ml polybrene. Two to four days after transduction, transduced GFP^+^ or mCherry^+^ cells were purified by flow cytometry.

### Immunohistochemistry

Paraffin-embedded sections (5 μm) were subjected to antigen retrieval using either 10 mM citrate buffer pH6 or Tris-EDTA buffer pH9, then blocked before staining with primary antibodies overnight at 4°C and incubation with a biotinylated secondary antibody at RT for 30 min. The streptavidin-based peroxidase detection system (ABC reagent, Vector Laboratories, Burlingame, CA) was used with 3,3-diaminobenzidine as substrate (DAKO, Glostrup, Denmark). In all cases, an isotype- matched control IgG was used as a negative control.

### Antibodies

For flow cytometry, antibodies against mouse antigens were purchased from Biolegend (San Diego, CA) or BD Biosciences (Franklin Lakes, NJ) unless otherwise specified and included CD24-PE (M1/69), CD31-B (MEC13.3), TER-119-B (TER-119), CD45-B (30-F11), CD29-FITC (HMB1-1), CD61-APC and streptavidin-APC-Cy7. For immunohistochemistry, the following antibodies were used: anti-SMA (1A4; Sigma), anti-p63 (4A4), K8/18 (Troma-1; Developmental Studies Hybridoma Bank, Iowa City), anti-Keratin 5 (Covance, Emeryville, CA), anti-Keratin 14 (LL002; Novocastra, UK), anti-E-cadherin, anti-ASAP1 (Rockland Immunochemicals Inc., Limerick, PA), anti-PROX1 (ab37128; Abcam, Cambridge, UK), anti-SNAI2 (#9585 Cell Signaling Technology, Massachussetts, USA). Secondary antibodies included biotin-conjugated goat anti-rabbit IgG, rabbit anti-rat IgG and goat anti-mouse IgG (Vector Laboratories, Burlingame, CA).

### shRNA amplicon sequencing and analysis

Genomic DNA was extracted using the DNeasy Blood kit (Qiagen) from samples taken at T0 and T2 timepoints and shRNA sequences were isolated from 200–800 μg gDNA (routinely 400 μg) using the PCR protocol outlined below. Primers were common to all shRNAs. The Forward primer, 5’-CAAGCAGAAGACGGCATACGAGCTCTTCCGATCTTAGTGAAGCCACAGATGTA-3’ anneals in the loop region and incorporates the P7 Illumina adapter sequence. The Reverse primer (5’-AATGATACGGCGACCACCGAGATCTACACTCTTTCCCTACACGACGCTCTTCCGATCTXXXXXGTAGCCCCTTGAATTCCGAG-3’) anneals in a region common to all shRNAs and incorporates a variable 5 bp index to enable multiplexing of samples, the P5 Illumina adapter sequence and the sequencing primer site. Following one round of PCR with an annealing temperature of 52°C and a further 30 to 32 cycles with an annealing temperature of 55°C, indexed, half shRNA products (168 bp) were pooled and sequenced on an Illumina GAIIx or HiSeq2000. Processing of the raw sequence reads was carried out in R as described previously [38]. Briefly, the number of perfect matches for each index-hairpin combination was tallied to give counts for the relevant hairpins in each sample. Downstream statistical analysis of the summarized counts was performed using the edgeR software (version 2.6.3) [44]. Outlier samples determined by visual inspection of multidimensional scaling plots were removed. An exact test for differences between the T14 and T2 biological replicate samples in each pool of shRNA was performed assuming a negative binomial distribution of the counts [45] and a common dispersion estimate. Log2-fold-changes, p-values and false discovery rates for each shRNA were reported.

### RNA-seq analysis

Total RNA was extracted and purified from: (1) sorted luminal or basal populations from the mammary glands of female virgin 8–10 week-old FVB/N mice (three independent samples for population), (2) MaSC/basal cells cultured for 1 week under mammosphere conditions, and (3) Comma Dβ cells grown under maintenance conditions [23]. Total RNA (100 ng) was used to generate sequencing libraries for whole transcriptome analysis following the Illumina’s TruSeq RNA v2 sample preparation protocol. Completed libraries were sequenced on HiSeq 2000 with TruSeq SBS Kit v3- HS reagents (Illumina) as 100 bp single-end reads at the Australian Genome Research Facility (AGRF), Melbourne. An average of 62 million 100 bp single-end reads were obtained per sample. Reads were aligned to the mouse reference genome (mm10) using the Rsubread package (version 1.14.1) [46] and assigned to genes using the featureCounts method [47]. Data were TMM normalized [48] and transformed into log2 counts per million. Linear models with observational-level weights [49] were fitted to obtain average expression values for each gene in each sample type and moderated t-statistics were used to assess differential expression between populations [50] using the limma package (version 3.20.5) [51]. False discovery rates [52] were used together with log2-fold-changes to rank genes. These data are available through GEO Series accession number GSE63310.

For comparison of gene expression across platforms, previously published microarray data (GSE19446) [24] were compared with RNA-seq profiles. Where multiple probes were available for a given gene, the probe with the highest average expression level was taken as representative. Genes were matched between platforms using gene symbols and respective log2-fold changes were plotted.

### Statistical analysis

Statistical analysis was performed using GraphPad Prism software (GraphPad, San Diego, CA). Data are shown as mean ± standard error of the mean (S.E.M.) or standard deviation (S.D.), where appropriate. Where applicable, the Student’s *t*-test was used, with p < 0.05 considered statistically significant.

## Additional file**s**

Additional file 1: Figure S1. MaSC/basal-derived mammosphere culture is permissive for the retention of progenitor and stem cell activity. (A) MaSC/basal cells cultured under mammosphere conditions generate multiple colony types including those with a luminal acinar morphology in Matrigel (arrowheads) Scale bar, 1 mm. (B) A carmine-alum stained whole-mounted mammary epithelial tree formed following transplantation of 500 MaSC/basal-derived mammosphere cells into a mammary fat pad pre-cleared of endogenous epithelium. (C) Table of limiting dilution analysis of transplanted mammosphere cells.
Additional file 2: Figure S2. Comparison between RNA-Seq expression profiles of primary mammary epithelial cell populations with the Illumina MouseWG-6 v2.0 BeadChip dataset. Plots showing differential expression of genes in the MaSC/basal versus luminal progenitor (LP) subset, MaSC/basal versus mature luminal (ML) cell subset and LP versus ML subset, as determined by microarray [24] and RNA-Seq. Genes differentially expressed with log2-fold-change > 2 and FDR < 0.001 were enumerated with those identifiable by both platforms highlighted in blue, RNA-Seq-only in yellow and microarray-only in red.
Additional file 3: Table S1. Table of shRNA clones included in the screen.
Additional file 4: Figure S3. *Snai2* is a positive regulator of mammary epithelial progenitor cells *in vitro.* (A) qRT-PCR detection of *Snai2* transcript abundance in MaSC/basal cells following transduction with retroviruses expressing sh*Control* or shRNAs targeting *Snai2*. Data are shown as mean ± S.D. *Snai2* expression normalized to *Gapdh* expression is shown relative to sh*Control* (n > 3). (B) Western blot analysis of Snai2 protein in MaSC/basal cells following transduction with retroviruses expressing sh*Control* or shRNAs targeting *Snai2*. (C) Transduced cells (500) were plated on an i3T3 feeder layer and cultured for 6 days to allow the formation of colonies. Left panel: histogram showing the number of colonies derived from cells transduced with sh*Control*- and sh*Snai2*-retroviruses. Data are shown as mean ± S.D for three independent experiments. Right panel: representative images of observed colonies. Scale bar, 0.5 cm.
