# Supplementary figures and images for: A pooled shRNA screen for regulators of primary mammary stem and progenitor cells identifies roles for Asap1 and Prox1
Source: BMC Cancer. 2015 Apr 3;15:221. doi: 10.1186/s12885-015-1187-z (PMC4399223; doi:10.1186/s12885-015-1187-z)

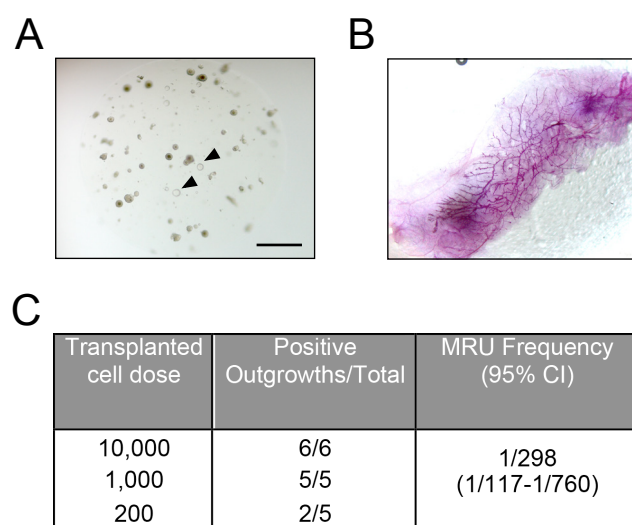

Supplement: Additional file 1: Figure S1. — MaSC/basal-derived mammosphere culture is permissive for the retention of progenitor and stem cell activity. (A) MaSC/basal cells cultured under mammosphere conditions generate multiple colony types including those with a luminal acinar morphology in Matrigel (arrowheads) Scale bar, 1 mm. (B) A carmine-alum stained whole-mounted mammary epithelial tree formed following transplantation of 500 MaSC/basal-derived mammosphere cells into a mammary fat pad pre-cleared of endogenous epithelium. (C) Table of limiting dilution analysis of transplanted mammosphere cells. [file 12885_2015_1187_MOESM1_ESM.pdf]

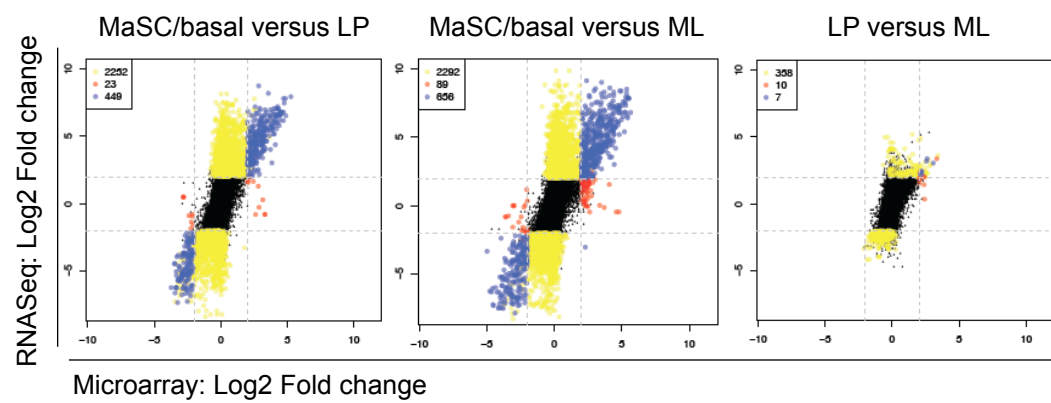

Supplement: Additional file 2: Figure S2. — Comparison between RNA-Seq expression profiles of primary mammary epithelial cell populations with the Illumina MouseWG-6 v2.0 BeadChip dataset. Plots showing differential expression of genes in the MaSC/basal versus luminal progenitor (LP) subset, MaSC/basal versus mature luminal (ML) cell subset and LP versus ML subset, as determined by microarray [24] and RNA-Seq. Genes differentially expressed with log2-fold-change > 2 and FDR < 0.001 were enumerated with those identifiable by both platforms highlighted in blue, RNA-Seq-only in yellow and microarray-only in red. [file 12885_2015_1187_MOESM2_ESM.pdf]

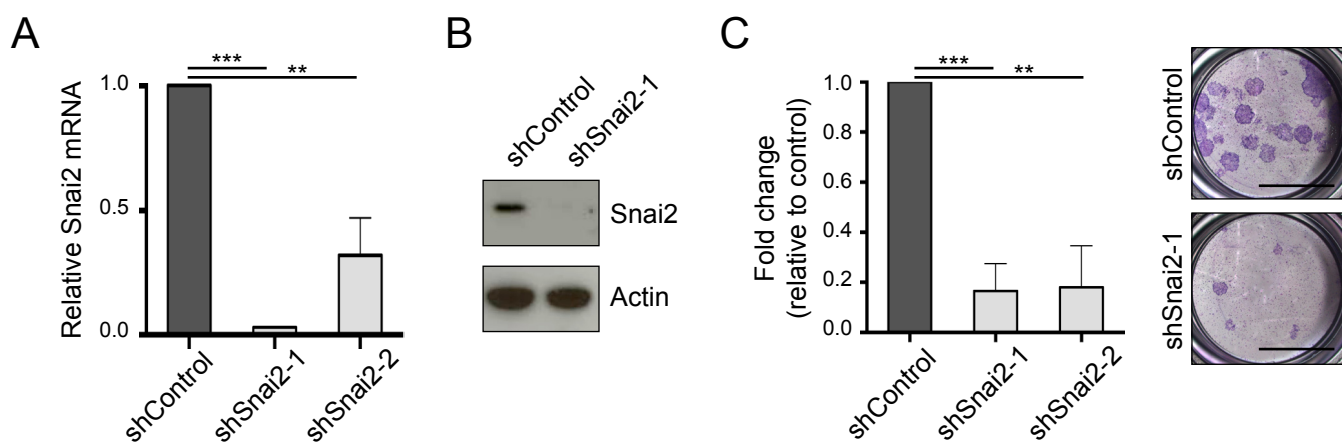

Supplement: Additional file 4: Figure S3. — Snai2 is a positive regulator of mammary epithelial progenitor cells in vitro. (A) qRT-PCR detection of Snai2 transcript abundance in MaSC/basal cells following transduction with retroviruses expressing shControl or shRNAs targeting Snai2. Data are shown as mean ± S.D. Snai2 expression normalized to Gapdh expression is shown relative to shControl (n > 3). (B) Western blot analysis of Snai2 protein in MaSC/basal cells following transduction with retroviruses expressing shControl or shRNAs targeting Snai2. (C) Transduced cells (500) were plated on an i3T3 feeder layer and cultured for 6 days to allow the formation of colonies. Left panel: histogram showing the number of colonies derived from cells transduced with shControl- and shSnai2-retroviruses. Data are shown as mean ± S.D for three independent experiments. Right panel: representative images of observed colonies. Scale bar, 0.5 cm. [file 12885_2015_1187_MOESM4_ESM.pdf]
