# Supplementary material for: A pooled shRNA screen for regulators of primary mammary stem and progenitor cells identifies roles for Asap1 and Prox1
Source: BMC Cancer. 2015 Apr 3;15:221. doi: 10.1186/s12885-015-1187-z (PMC4399223; doi:10.1186/s12885-015-1187-z)
Supplement: Additional file 3: Table S1. — Table of shRNA clones included in the screen. [file 12885_2015_1187_MOESM3_ESM.docx]

**Additional file 3: Table S1. Table of shRNA clones included in the screen.**

| Vendor CloneID | Gene symbol |
| --- | --- |
| V2LMM_6871 | MYOG |
| V2LMM_68084 | DDEF1 |
| V2LMM_63192 | LASS6 |
| V2LMM_64405 | NR0B1 |
| V2LMM_189334 | SOX21 |
| V2LMM_82812 | ID1 |
| V2LMM_73029 | Myst1 |
| V2LMM_62263 | TBP |
| V2LMM_25195 | TCEA2 |
| V2LMM_13506 | NKX3-1 |
| V2LMM_54422 | POU1F1 |
| V2LMM_9553 | FOXN1 |
| V2LMM_259235 | FOXC2 |
| V2LMM_53719 | TEF |
| V2LMM_27467 | NFE2L1 |
| V2LMM_937 | MNT |
| V2LMM_20404 | MAFG |
| V2LMM_218286 | SOX9 |
| V2LMM_29411 | EZH2 |
| V2LMM_64907 | NR1H3 |
| V2LMM_226932 | NOTCH2 |
| V2LMM_22166 | NR1H4 |
| V2LMM_70598 | KLF4 |
| V2LMM_29885 | LMO2 |
| V2LMM_85045 | ZFP449 |
| V2LMM_27178 | TRPS1 |
| V2LMM_7277 | NRARP |
| V2LMM_71087 | LEF1 |
| V2LMM_37331 | OVOL2 |
| V2LMM_82311 | FOXJ1 |
| V2LMM_30422 | EZH2 |
| V2LMM_86479 | HLF |
| V2LMM_73715 | KLF5 |
| V2LMM_77459 | TCF4 |
| V2LMM_87315 | ANKRD46 |
| V2LMM_249987 | ANKRD33 |
| V2LMM_34449 | THRB |
| V2LMM_34394 | MRG2 |
| V2LMM_71843 | TCF19 |
| V2LMM_162758 | ETS1 |
| V2LMM_103225 | Rbbp8 |
| V2LMM_3550 | TCF21 |
| V2LMM_61869 | TBP |
| V2LMM_110117 | NFATC3 |
| V2LMM_70871 | NR1I3 |
| V2LMM_196851 | GATA6 |
| V2LMM_86811 | Klf8 |
| V2LMM_14204 | Cbx4 |
| V2LMM_103224 | Rbbp8 |
| V2LMM_2021 | MTA3 |
| V2LMM_18790 | CLOCK |
| V2LMM_8902 | 2410018C20RIK |
| V2LMM_7952 | NR2C1 |
| V2LMM_65378 | E2F6 |
| V2LMM_14131 | Id3 |
| V2LMM_4616 | Cbx4 |
| V2LMM_81587 | Klf9 |
| V2LMM_50581 | NR2F2 |
| V2LMM_71013 | HNRPAB |
| V2LMM_71313 | CML3 |
| V2LMM_46526 | PROX1 |
| V2LMM_63443 | MAFB |
| V2LMM_75410 | ASB4 |
| V2LMM_87318 | ANKRD46 |
| V2LMM_88650 | 6430502M16RIK |
| V2LMM_194038 | HOXD8 |
| V2LMM_71230 | NR1I3 |
| V2LMM_79666 | PAX8 |
| V2LMM_64571 | GCDH |
| V2LMM_50262 | VDR |
| V2LMM_66887 | LASS4 |
| V2LMM_71060 | GTF2H4 |
| V2LMM_5760 | MYC |
| V2LMM_6641 | PYCARD |
| V2LMM_29358 | TBX20 |
| V2LMM_212338 | PPP1R16B |
| V2LMM_68781 | OTX1 |
| V2LMM_71737 | HOXA5 |
| V2LMM_67978 | LASS4 |
| V2LMM_62940 | IRX6 |
| V2LMM_11432 | CITED2 |
| V2LMM_46319 | Atf4 |
| V2LMM_7663 | ZSCAN21 |
| V2LMM_78900 | ARNTL |
| V2LMM_74890 | ANKFY1 |
| V2LMM_75524 | SIX6 |
| V2LMM_70474 | Brca1 |
| V2LMM_48343 | NR2F2 |
| V2LMM_19589 | Smad2 |
| V2LMM_61743 | Tcf4 |
| V2LMM_6125 | ANKRD32 |
| V2LMM_18658 | IRX5 |
| V2LMM_78314 | NR1H3 |
| V2LMM_16001 | Klf3 |
| V2LMM_73750 | Brca2 |
| V2LMM_74023 | PITX2 |
| V2LMM_111855 | GLI2 |
| V2LMM_91370 | BSX |
| V2LMM_53426 | TGIF2 |
| V2LMM_116867 | CEBPE |
| V2LMM_128917 | ZKSCAN2 |
| V2LMM_17497 | CDX2 |
| V2LMM_82986 | PAX7 |
| V2LMM_15471 | HOXB13 |
| V2LMM_70426 | Brca1 |
| V2LMM_11891 | CEBPA |
| V2LMM_63183 | MGA |
| V2LMM_79243 | GATA3 |
| V2LMM_17562 | NFYA |
| V2LMM_7775 | MTA3 |
| V2LMM_215563 | RXRA |
| V2LMM_221541 | TBP |
| V2LMM_15414 | Smad2 |
| V2LMM_76848 | BARX2 |
| V2LMM_68220 | HOXD1 |
| V2LMM_64847 | RUNX1T1 |
| V2LMM_108735 | SIX5 |
| V2LMM_45655 | ESR2 |
| V2LMM_36448 | TBX18 |
| V2LMM_42543 | OBOX3 |
| V2LMM_205971 | NR3C2 |
| V2LMM_2215 | Cbx5 |
| V2LMM_74031 | Id2 |
| V2LMM_176254 | FOXP1 |
| V2LMM_10928 | SOX13 |
| V2LMM_70731 | KLF16 |
| V2LMM_40150 | FOXC1 |
| V2LMM_52067 | PGR |
| V2LMM_188297 | EOMES |
| V2LMM_193883 | SPIB |
| V2LMM_207277 | ZHX2 |
| V2LMM_57548 | BAT4 |
| V2LMM_12423 | ANKRD32 |
| V2LMM_96807 | TANC2 |
| V2LMM_37580 | TAF7 |
| V2LMM_68161 | ANKZF1 |
| V2LMM_69021 | ZKSCAN6 |
| V2LMM_71278 | HEY2 |
| V2LMM_56476 | ALX1 |
| V2LMM_9074 | MYO3A |
| V2LMM_12510 | Snai2 |
| V2LMM_190690 | POU2F1 |
| V2LMM_20489 | PHF5A |
| V2LMM_49944 | CREB3 |
| V2LMM_217947 | SOX4 |
| V2LMM_51155 | IKZF1 |
| V2LMM_75154 | HOXD3 |
| V2LMM_86857 | HELT |
| V2LMM_244811 | PPARD |
| V2LMM_216290 | Notch4 |
| V2LMM_73187 | HEY1 |
| V2LMM_7544 | FANK1 |
| V2LMM_64645 | Brca2 |
| V2LMM_218874 | PHOX2A |
| V2LMM_16617 | ZFP628 |
| V2LMM_83177 | TCEA3 |
| V2LMM_74358 | RUNX2 |
| V2LMM_109364 | MAZ |
| V2LMM_360 | ONECUT1 |
| V2LMM_62292 | POU2F3 |
| V2LMM_33464 | E2F5 |
| V2LMM_76959 | POU6F2 |
| V2LMM_52090 | USF1 |
| V2LMM_8662 | SOX17 |
| V2LMM_78592 | SNAI3 |
| V2LMM_255260 | FOXC2 |
| V2LMM_7675 | CEBPA |
| V2LMM_193236 | OVOL2 |
| V2LMM_119565 | ADNP2 |
| V2LMM_153495 | ZHX2 |
| V2LMM_254400 | SCAND1 |
| V2LMM_63129 | MTF2 |
| V2LMM_92204 | TFDP2 |
| V2LMM_22132 | ELF3 |
| V2LMM_30793 | FOXC1 |
| V2LMM_111857 | GLI2 |
| V2LMM_69562 | ABTB1 |
| V2LMM_161871 | NFXL1 |
| V2LMM_68970 | RXRB |
| V2LMM_33687 | MRG2 |
| V2LMM_93460 | ANKRD15 |
| V2LMM_62864 | TRPV6 |
| V2LMM_72868 | RUNX2 |
| V2LMM_72461 | LASS4 |
| V2LMM_73846 | CCL5 |
| V2LMM_42520 | STAT2 |
| V2LMM_27362 | HSF4 |
| V2LMM_261712 | TBX10 |
| V2LMM_13334 | NFE2L3 |
| V2LMM_71058 | ANKZF1 |
| V2LMM_9341 | NFE2L3 |
| V2LMM_195956 | HOXC13 |
| V2LMM_66050 | FOXA2 |
| V2LMM_91876 | E2F7 |
| V2LMM_63160 | KLF12 |
| V2LMM_103491 | TSHZ1 |
| V2LMM_24280 | THRB |
| V2LMM_23696 | PAX1 |
| V2LMM_9596 | TWIST2 |
| V2LMM_7041 | NFKB2 |
| V2LMM_14620 | FOXA1 |
| V2LMM_150040 | PURB |
| V2LMM_46846 | ESR2 |
| V2LMM_11798 | Prdm1 |
| V2LMM_62162 | SIM1 |
| V2LMM_85041 | ZFP449 |
| V2LMM_15759 | HDAC2 |
| V2LMM_2692 | BACH2 |
| V2LMM_16555 | FOXQ1 |
| V2LMM_29896 | ARID3A |
| V2LMM_20599 | PAX1 |
| V2LMM_79660 | RARG |
| V2LMM_61521 | EN2 |
| V2LMM_90910 | AW146020 |
| V2LMM_3186 | FOXA1 |
| V2LMM_81466 | CML3 |
| V2LMM_52405 | ELK3 |
| V2LMM_75349 | MGA |
| V2LMM_24543 | ELK1 |
| V2LMM_69294 | POU2AF1 |
| V2LMM_7624 | RCOR2 |
| V2LMM_18889 | PYCARD |
| V2LMM_34873 | Smad5 |
| V2LMM_104865 | 4931423N10RIK |
| V2LMM_249066 | SCAND1 |
| V2LMM_69359 | HIF1A |
| V2LMM_166643 | ZKSCAN1 |
| V2LMM_159278 | IKZF3 |
| V2LMM_217187 | Id3 |
| V2LMM_73421 | CREB3L4 |
| V2LMM_13439 | DACH1 |
| V2LMM_88216 | TRPS1 |
| V2LMM_76766 | NR1I3 |
| V2LMM_2755 | TGIFX1 |
| V2LMM_16243 | LCOR |
| V2LMM_72281 | KLF5 |
| V2LMM_72721 | HOXD1 |
| V2LMM_22409 | EHF |
| V2LMM_82130 | FEM1C |
| V2LMM_20348 | Cbx4 |
| V2LMM_112698 | SATB2 |
| V2LMM_103492 | TSHZ1 |
| V2LMM_61137 | FOXF2 |
| V2LMM_71108 | GATAD1 |
| V2LMM_83128 | SP3 |
| V2LMM_3484 | FOXA3 |
| V2LMM_134180 | FOXK1 |
| V2LMM_29499 | NFXL1 |
| V2LMM_600 | Notch4 |
| V2LMM_57715 | MNX1 |
| V2LMM_24420 | TSHZ3 |
| V2LMM_25054 | ELF2 |
| V2LMM_39085 | TSC22D1 |
| V2LMM_37110 | ARID3A |
| V2LMM_150349 | POU5F2 |
| V2LMM_197067 | Klf8 |
| V2LMM_68260 | LHX9 |
| V2LMM_101144 | IRX2 |
| V2LMM_39364 | STAT6 |
| V2LMM_21583 | Lmo2 |
| V2LMM_12312 | IRX3 |
| V2LMM_9612 | HSF2 |
| V2LMM_31437 | SMAD1 |
| V2LMM_72813 | PCDHB16 |
| V2LMM_16040 | ETV3 |
| V2LMM_18329 | MAFK |
| V2LMM_20128 | HTR5A |
| V2LMM_6385 | SOX5 |
| V2LMM_26985 | OVOL1 |
| V2LMM_100770 | FOXN3 |
| V2LMM_66633 | ZKSCAN1 |
| V2LMM_59402 | UGP2 |
| V2LMM_44007 | NR4A2 |
| V2LMM_223027 | AFF1 |
| V2LMM_69393 | GATA1 |
| V2LMM_75653 | CREB3L4 |
| V2LMM_71167 | HEY2 |
| V2LMM_79141 | POU2AF1 |
| V2LMM_101148 | IRX2 |
| V2LMM_29327 | CLPB |
| V2LMM_11721 | TRPC3 |
| V2LMM_80227 | LEF1 |
| V2LMM_18886 | CLOCK |
| V2LMM_83125 | SOX8 |
| V2LMM_160396 | ANKHD1 |
| V2LMM_89069 | ZFP446 |
| V2LMM_17767 | Notch4 |
| V2LMM_77841 | Lmo4 |
| V2LMM_96805 | TANC2 |
| V2LMM_86815 | Klf8 |
| V2LMM_72559 | DBP |
| V2LMM_76118 | SIX6 |
| V2LMM_74251 | MGA |
| V2LMM_77662 | LHX9 |
| V2LMM_210579 | TBPL2 |
| V2LMM_35988 | Ezh2 |
| V2LMM_103223 | Rbbp8 |
| V2LMM_261527 | ZKSCAN5 |
| V2LMM_24477 | MEF2B |
| V2LMM_64458 | LHX3 |
| V2LMM_63795 | KLF16 |
| V2LMM_69811 | HOXB4 |
| V2LMM_78161 | RORA |
| V2LMM_78695 | GATA5 |
| V2LMM_196631 | TBX3 |
| V2LMM_806 | TCFEB |
| V2LMM_17817 | TCEA1 |
| V2LMM_66703 | JUN |
| V2LMM_32865 | NPAS2 |
| V2LMM_23805 | TCF7L2 |
| V2LMM_238681 | MXD4 |
| V2LMM_17725 | IRX4 |
| V2LMM_62052 | GSC |
| V2LMM_59444 | POU3F4 |
| V2LMM_74201 | POU2F2 |
| V2LMM_78165 | 4921520G13RIK |
| V2LMM_36809 | KRI1 |
| V2LMM_92655 | SS18L1 |
| V2LMM_51687 | ZHX1 |
| V2LMM_12135 | FOXD1 |
| V2LMM_55532 | CENTG3 |
| V2LMM_46815 | DLX3 |
| V2LMM_78516 | ATF1 |
| V2LMM_246137 | ANKRA2 |
| V2LMM_83163 | TBX3 |
| V2LMM_69074 | FOXL1 |
| V2LMM_11958 | HOXA13 |
| V2LMM_73179 | LHX4 |
| V2LMM_25325 | Ezh2 |
| V2LMM_53535 | GBX2 |
| V2LMM_32418 | SMAD1 |
| V2LMM_87319 | ANKRD46 |
| V2LMM_160531 | OVOL1 |
| V2LMM_1085 | PYCARD |
| V2LMM_11490 | TRPC3 |
| V2LMM_75632 | 4921520G13RIK |
| V2LMM_82868 | LHX8 |
| V2LMM_91880 | E2F7 |
| V2LMM_47700 | PHOX2B |
| V2LMM_84597 | GPBP1 |
| V2LMM_195762 | MEOX2 |
| V2LMM_30526 | CEBPG |
| V2LMM_71273 | IRF4 |
| V2LMM_2543 | STAT4 |
| V2LMM_153813 | NFIA |
| V2LMM_78673 | HIVEP2 |
| V2LMM_84932 | A930001N09RIK |
| V2LMM_161870 | NFXL1 |
| V2LMM_9060 | 2410018C20RIK |
| V2LMM_56204 | VDR |
| V2LMM_82949 | NR1I2 |
| V2LMM_69360 | GATA3 |
| V2LMM_246124 | HOXB1 |
| V2LMM_82329 | HSF1 |
| V2LMM_41141 | FOXF2 |
| V2LMM_63548 | GABPA |
| V2LMM_31448 | NFIC |
| V2LMM_43136 | NOBOX |
| V2LMM_47011 | NFIA |
| V2LMM_72699 | 1810007M14RIK |
| V2LMM_31782 | ESRRG |
| V2LMM_62457 | CUTL1 |
| V2LMM_65643 | HIVEP2 |
| V2LMM_57665 | EHMT1 |
| V2LMM_37087 | SP4 |
| V2LMM_79080 | HOXB8 |
| V2LMM_66848 | RUNX1T1 |
| V2LMM_74274 | MIXL1 |
| V2LMM_70164 | CCL5 |
| V2LMM_7596 | CDX4 |
| V2LMM_44368 | EN2 |
| V2LMM_94322 | A030003K21RIK |
| V2LMM_180521 | Klf6 |
| V2LMM_72842 | HOXB7 |
| V2LMM_74634 | Id2 |
| V2LMM_62333 | RUNX1T1 |
| V2LMM_28115 | E2F1 |
| V2LMM_23644 | NFKBIE |
| V2LMM_34930 | FOXC1 |
| V2LMM_3898 | FOXP1 |
| V2LMM_50385 | PROP1 |
| V2LMM_4839 | Prdm1 |
| V2LMM_34512 | RUNX3 |
| V2LMM_64978 | LHX2 |
| V2LMM_2638 | TBX6 |
| V2LMM_3279 | ZKSCAN14 |
| V2LMM_63792 | CREB1 |
| V2LMM_101991 | SREBF2 |
| V2LMM_41753 | RHOX4B |
| V2LMM_66655 | NR2E3 |
| V2LMM_59073 | GRHL1 |
| V2LMM_29882 | TSHZ3 |
| V2LMM_81364 | ALX3 |
| V2LMM_8341 | TRIM28 |
| V2LMM_99409 | BTBD11 |
| V2LMM_7452 | HDAC1 |
| V2LMM_63211 | HEY1 |
| V2LMM_85044 | ZFP449 |
| V2LMM_21386 | WT1 |
| V2LMM_82321 | HOXB9 |
| V2LMM_72990 | RARB |
| V2LMM_82314 | FOXJ1 |
| V2LMM_125912 | CBFA2T2 |
| V2LMM_73393 | HOXA1 |
| V2LMM_21246 | ANKRD22 |
| V2LMM_8888 | MEOX1 |
| V2LMM_31304 | ANKRD5 |
| V2LMM_89558 | MYST2 |
| V2LMM_2989 | ZFP287 |
| V2LMM_2898 | NFKB2 |
| V2LMM_232412 | NFE2 |
| V2LMM_69144 | ARNT2 |
| V2LMM_150209 | ZBTB24 |
| V2LMM_6881 | RARA |
| V2LMM_82318 | HOXB9 |
| V2LMM_191393 | AFF3 |
| V2LMM_64026 | XBP1 |
| V2LMM_874 | TBX21 |
| V2LMM_96806 | TANC2 |
| V2LMM_50956 | TGIF2 |
| V2LMM_66535 | FOXP4 |
| V2LMM_193365 | DLX1 |
| V2LMM_26775 | IKZF2 |
| V2LMM_16657 | SOX5 |
| V2LMM_77443 | PAX9 |
| V2LMM_31989 | E2F5 |
| V2LMM_13644 | POLR3K |
| V2LMM_3894 | TBX1 |
| V2LMM_53631 | CTBP1 |
| V2LMM_88780 | ZFP367 |
| V2LMM_5179 | NR5A2 |
| V2LMM_78823 | PCDHB16 |
| V2LMM_22804 | SOX21 |
| V2LMM_73432 | HIVEP2 |
| V2LMM_82328 | HSF1 |
| V2LMM_133345 | MGA |
| V2LMM_36532 | ANKRD36 |
| V2LMM_70538 | Myst1 |
| V2LMM_11882 | ANKRD32 |
| V2LMM_65210 | HNF4G |
| V2LMM_92824 | BCORL1 |
| V2LMM_15426 | TEAD2 |
| V2LMM_2630 | Id3 |
| V2LMM_20872 | NOTCH1 |
| V2LMM_76275 | CDX1 |
| V2LMM_26296 | NFYC |
| V2LMM_30823 | TCFAP2B |
| V2LMM_224863 | FKHL18 |
| V2LMM_4301 | E2F3 |
| V2LMM_23617 | MYT1 |
| V2LMM_65193 | PPARG |
| V2LMM_94526 | HOMEZ |
| V2LMM_77185 | ATF3 |
| V2LMM_16984 | CREBZF |
| V2LMM_253355 | ELF1 |
| V2LMM_13100 | SOX6 |
| V2LMM_74784 | ATM |
| V2LMM_59203 | PHOX2B |
| V2LMM_9354 | TBX22 |
| V2LMM_3301 | HNF4A |
| V2LMM_14560 | HTR5A |
| V2LMM_71334 | GABPA |
| V2LMM_89553 | MYST2 |
| V2LMM_19796 | Klf3 |
| V2LMM_46076 | PREB |
| V2LMM_33650 | ESRRG |
| V2LMM_4942 | MAFF |
| V2LMM_22164 | ZFP110 |
| V2LMM_222875 | POU6F1 |
| V2LMM_177350 | MAF |
| V2LMM_60544 | TSC22D3 |
| V2LMM_226793 | CPHX |
| V2LMM_13307 | TRIM28 |
| V2LMM_56821 | ASB15 |
| V2LMM_82987 | PAX7 |
| V2LMM_78277 | RXRA |
| V2LMM_70600 | TCF20 |
| V2LMM_57315 | AHR |
| V2LMM_153816 | NFIA |
| V2LMM_50004 | DLX3 |
| V2LMM_25660 | NR1D1 |
| V2LMM_81315 | FOXD4 |
| V2LMM_26177 | TCFAP2B |
| V2LMM_82049 | SIM1 |
| V2LMM_212428 | CREB3L2 |
| V2LMM_2195 | FOXO4 |
| V2LMM_16708 | CTNNB1 |
| V2LMM_71498 | FOXA2 |
| V2LMM_134421 | CREB3L2 |
| V2LMM_87896 | ANKRD56 |
| V2LMM_65781 | HOXD4 |
| V2LMM_3018 | SOX9 |
| V2LMM_161872 | NFXL1 |
| V2LMM_188421 | HOXC10 |
| V2LMM_225487 | MKX |
| V2LMM_68103 | GATAD1 |
| V2LMM_3277 | HNF4A |
| V2LMM_9444 | BARD1 |
| V2LMM_122258 | HDX |
| V2LMM_72122 | ATF3 |
| V2LMM_93490 | GTF2H3 |
| V2LMM_26624 | SRF |
| V2LMM_84077 | TAF11 |
| V2LMM_73731 | ATF3 |
| V2LMM_58771 | NR2F2 |
| V2LMM_65058 | RORC |
| V2LMM_38933 | ZBTB48 |
| V2LMM_12899 | Snai2 |
| V2LMM_79308 | Klf10 |
| V2LMM_20822 | Lmo2 |
| V2LMM_20637 | ANKRD33 |
| V2LMM_25925 | NFYC |
| V2LMM_134422 | CREB3L2 |
| V2LMM_41597 | MZF1 |
| V2LMM_13696 | STAT3 |
| V2LMM_71488 | GTF2I |
| V2LMM_2247 | STAT4 |
| V2LMM_74434 | HOXA1 |
| V2LMM_1274 | NR1D2 |
| V2LMM_45878 | TAF5 |
| V2LMM_63806 | Brca2 |
| V2LMM_63726 | TRPV6 |
| V2LMM_27383 | ZFP189 |
| V2LMM_93464 | ANKRD15 |
| V2LMM_69972 | LMX1B |
| V2LMM_208988 | TCF7L2 |
| V2LMM_195918 | GBX1 |
| V2LMM_65751 | RARB |
| V2LMM_20788 | TAF6L |
| V2LMM_25678 | TITF1 |
| V2LMM_46558 | POU3F3 |
| V2LMM_125916 | CBFA2T2 |
| V2LMM_68498 | MIXL1 |
| V2LMM_81594 | CECR6 |
| V2LMM_46239 | ANKRD42 |
| V2LMM_73666 | ASB5 |
| V2LMM_43299 | FOXJ3 |
| V2LMM_75183 | E2F2 |
| V2LMM_48075 | VDR |
| V2LMM_6192 | SOX7 |
| V2LMM_120456 | BTF3 |
| V2LMM_88649 | 6430502M16RIK |
| V2LMM_55158 | BAT4 |
| V2LMM_20679 | CLPB |
| V2LMM_24208 | ESRRB |
| V2LMM_3123 | KLF2 |
| V2LMM_10461 | ZKSCAN14 |
| V2LMM_244133 | IRX4 |
| V2LMM_611 | SOX7 |
| V2LMM_20896 | NKX2-5 |
| V2LMM_50871 | NFIL3 |
| V2LMM_101147 | IRX2 |
| V2LMM_62330 | RARG |
| V2LMM_197604 | ANKIB1 |
| V2LMM_174528 | GATAD2B |
| V2LMM_162662 | ZFHX3 |
| V2LMM_164864 | ZKSCAN1 |
| V2LMM_30371 | NR0B2 |
| V2LMM_109356 | GTF3C1 |
| V2LMM_62680 | HNF4G |
| V2LMM_77526 | HOXC6 |
| V2LMM_63982 | POU6F2 |
| V2LMM_676 | SPIC |
| V2LMM_104864 | 4931423N10RIK |
| V2LMM_70199 | RUNX1 |
| V2LMM_64308 | NR2E3 |
| V2LMM_57226 | EN1 |
| V2LMM_36402 | PAX3 |
| V2LMM_57535 | TNNI3K |
| V2LMM_177575 | RORA |
| V2LMM_3372 | LMO1 |
| V2LMM_5763 | VSX1 |
| V2LMM_16894 | LASS5 |
| V2LMM_70597 | LHX4 |
| V2LMM_61672 | RORA |
| V2LMM_112696 | SATB2 |
| V2LMM_55515 | DLX2 |
| V2LMM_52432 | EPAS1 |
| V2LMM_63208 | HOXD3 |
| V2LMM_45542 | ANKRD10 |
| V2LMM_30259 | FBXW7 |
| V2LMM_22904 | DMRTA1 |
| V2LMM_10406 | MNT |
| V2LMM_111380 | ZSCAN2 |
| V2LMM_29891 | PRRX1 |
| V2LMM_52149 | NR2F1 |
| V2LMM_62571 | ATF3 |
| V2LMM_355 | ASB6 |
| V2LMM_57461 | FOS |
| V2LMM_63095 | TRPC4 |
| V2LMM_20115 | CDKN2C |
| V2LMM_9544 | HOXB13 |
| V2LMM_31682 | ZFP354A |
| V2LMM_4549 | NFIC |
| V2LMM_72857 | PFDN1 |
| V2LMM_55490 | YY1 |
| V2LMM_69443 | CDKN2D |
| V2LMM_119568 | ADNP2 |
| V2LMM_66519 | FOXP4 |
| V2LMM_53365 | TRPC5 |
| V2LMM_37033 | NFATC4 |
| V2LMM_171535 | ARNTL2 |
| V2LMM_122256 | HDX |
| V2LMM_63901 | FOSL2 |
| V2LMM_10428 | REL |
| V2LMM_37078 | NPAS2 |
| V2LMM_81398 | NR2E3 |
| V2LMM_169814 | GPBP1 |
| V2LMM_216697 | NRL |
| V2LMM_247448 | LHX5 |
| V2LMM_6723 | IRF8 |
| V2LMM_64974 | Klf10 |
| V2LMM_23343 | MYCN |
| V2LMM_25165 | GTF2B |
| V2LMM_189815 | KLF11 |
| V2LMM_62715 | ARNT2 |
| V2LMM_4633 | SOX7 |
| V2LMM_63029 | SPZ1 |
| V2LMM_31438 | NR1D1 |
| V2LMM_56134 | NFXL1 |
| V2LMM_59503 | TAF5 |
| V2LMM_48681 | MSX2 |
| V2LMM_22018 | ELK4 |
| V2LMM_210351 | HIF1A |
| V2LMM_67917 | RUNX2 |
| V2LMM_34013 | MTF1 |
| V2LMM_219405 | PURB |
| V2LMM_80478 | HESX1 |
| V2LMM_74491 | POU5F1 |
| V2LMM_1090 | CTNNB1 |
| V2LMM_218988 | PURB |
| V2LMM_3897 | DDIT3 |
| V2LMM_51702 | TSC22D3 |
| V2LMM_4646 | NFAT5 |
| V2LMM_65067 | GCDH |
| V2LMM_219690 | POU4F2 |
| V2LMM_30632 | NFIB |
| V2LMM_12514 | MTA1 |
| V2LMM_44071 | ZFP445 |
| V2LMM_70754 | CML3 |
| V2LMM_188386 | NFE2 |
| V2LMM_168134 | GATAD1 |
| V2LMM_65893 | PPARA |
| V2LMM_12458 | NKX3-1 |
| V2LMM_80930 | DDEF1 |
| V2LMM_48001 | ASB15 |
| V2LMM_195291 | FEM1C |
| V2LMM_63529 | PAX9 |
| V2LMM_105135 | HOXD8 |
| V2LMM_150041 | PURB |
| V2LMM_225826 | AW146020 |
| V2LMM_68681 | PITX1 |
| V2LMM_162661 | ZFHX3 |
| V2LMM_58693 | IRF7 |
| V2LMM_50091 | NFXL1 |
| V2LMM_82872 | LHX8 |
| V2LMM_79134 | TCF20 |
| V2LMM_38268 | MEF2D |
| V2LMM_205569 | EOMES |
| V2LMM_64023 | LEF1 |
| V2LMM_71347 | E2F2 |
| V2LMM_87899 | ANKRD56 |
| V2LMM_54583 | CIAO1 |
| V2LMM_13047 | TCEA1 |
| V2LMM_55395 | CIAO1 |
| V2LMM_78253 | ASB8 |
| V2LMM_61682 | ZFHX3 |
| V2LMM_195814 | ANKRD56 |
| V2LMM_38800 | NR0B2 |
| V2LMM_153514 | POU6F1 |
| V2LMM_79163 | HOXB4 |
| V2LMM_72487 | ZKSCAN6 |
| V2LMM_218221 | ELK4 |
| V2LMM_39208 | NFKB1 |
| V2LMM_34724 | SPEN |
| V2LMM_243099 | HOXB1 |
| V2LMM_94625 | FOXI2 |
| V2LMM_45418 | CARF |
| V2LMM_17865 | MLX |
| V2LMM_69631 | HOXA11 |
| V2LMM_24922 | ANKRD49 |
| V2LMM_56702 | USF1 |
| V2LMM_250640 | ZBTB24 |
| V2LMM_22535 | ANKRD33 |
| V2LMM_206283 | NR3C2 |
| V2LMM_62241 | HESX1 |
| V2LMM_159276 | IKZF3 |
| V2LMM_8836 | CASKIN1 |
| V2LMM_163469 | ANKAR |
| V2LMM_50313 | ANKRD42 |
| V2LMM_196824 | IRF6 |
| V2LMM_191881 | BCOR |
| V2LMM_32733 | MYF6 |
| V2LMM_24569 | ARID3A |
| V2LMM_4829 | MAFK |
| V2LMM_71197 | HNRPAB |
| V2LMM_64065 | CUTL1 |
| V2LMM_64311 | SP1 |
| V2LMM_22551 | SEBOX |
| V2LMM_217748 | GATA6 |
| V2LMM_74500 | POU6F2 |
| V2LMM_81621 | SIX6 |
| V2LMM_33018 | E2F5 |
| V2LMM_9441 | TBX6 |
| V2LMM_18049 | SPIC |
| V2LMM_4544 | CDKN2C |
| V2LMM_8632 | TWIST1 |
| V2LMM_82370 | MEOX2 |
| V2LMM_188405 | HOXC10 |
| V2LMM_43519 | CREBL1 |
| V2LMM_226585 | MEF2D |
| V2LMM_49897 | NFIA |
| V2LMM_73046 | HMX1 |
| V2LMM_153493 | ZHX2 |
| V2LMM_23763 | THRB |
| V2LMM_83105 | SIX4 |
| V2LMM_50813 | FOSL1 |
| V2LMM_12139 | TGIFX1 |
| V2LMM_190516 | TBX3 |
| V2LMM_52266 | EMX2 |
| V2LMM_12338 | NKX2-2 |
| V2LMM_29928 | PAX3 |
| V2LMM_4298 | HOXA13 |
| V2LMM_73821 | NCOR1 |
| V2LMM_63839 | ARNTL |
| V2LMM_128920 | ZKSCAN2 |
| V2LMM_16290 | MEIS1 |
| V2LMM_237428 | ZBTB24 |
| V2LMM_7872 | MAFK |
| V2LMM_62034 | SHOX2 |
| V2LMM_26709 | ASB10 |
| V2LMM_66412 | NCOR1 |
| V2LMM_242512 | TBPL2 |
| V2LMM_37226 | NFKB1 |
| V2LMM_22603 | E2F5 |
| V2LMM_76180 | MTF2 |
| V2LMM_67088 | CEBPB |
| V2LMM_75902 | PA2G4 |
| V2LMM_160680 | AFF3 |
| V2LMM_217646 | RBPJL |
| V2LMM_37491 | ZKSCAN1 |
| V2LMM_19625 | FOXP1 |
| V2LMM_18443 | NR3C1 |
| V2LMM_94626 | FOXI2 |
| V2LMM_63476 | PITX2 |
| V2LMM_83168 | TBX5 |
| V2LMM_95401 | MEF2D |
| V2LMM_197368 | FEM1C |
| V2LMM_96808 | TANC2 |
| V2LMM_30824 | SMAD1 |
| V2LMM_82740 | GATA6 |
| V2LMM_61755 | CCL5 |
| V2LMM_1840 | HOXB5 |
| V2LMM_93313 | TANC2 |
| V2LMM_136277 | ESR1 |
| V2LMM_61844 | PPARG |
| V2LMM_220263 | CECR6 |
| V2LMM_75471 | Klf9 |
| V2LMM_108488 | ANKRD7 |
| V2LMM_82785 | HOXC13 |
| V2LMM_19804 | CDX4 |
| V2LMM_30015 | OVOL2 |
| V2LMM_4816 | SPIC |
| V2LMM_177578 | RORA |
| V2LMM_37413 | TEAD1 |
| V2LMM_57985 | NR2F1 |
| V2LMM_9971 | ZFP287 |
| V2LMM_6131 | MEIS1 |
| V2LMM_52610 | POU1F1 |
| V2LMM_68316 | LBX1 |
| V2LMM_63694 | E2F2 |
| V2LMM_9571 | MYO3A |
| V2LMM_55159 | ELF5 |
| V2LMM_110972 | LBA1 |
| V2LMM_55861 | ISL1 |
| V2LMM_134183 | FOXK1 |
| V2LMM_84596 | GPBP1 |
| V2LMM_67752 | CDKN2D |
| V2LMM_33702 | HSF4 |
| V2LMM_132837 | GLIS3 |
| V2LMM_244670 | TLX1 |
| V2LMM_30190 | TBX18 |
| V2LMM_195610 | LHX8 |
| V2LMM_6865 | CDX2 |
| V2LMM_92659 | SS18L1 |
| V2LMM_65089 | HOXD3 |
| V2LMM_16424 | DDIT3 |
| V2LMM_81503 | Brca2 |
| V2LMM_13729 | TLE4 |
| V2LMM_77683 | HOXD3 |
| V2LMM_224991 | MKX |
| V2LMM_25613 | TFAM |
| V2LMM_51583 | AR |
| V2LMM_43391 | SOX30 |
| V2LMM_254123 | ANKRD54 |
| V2LMM_9506 | GSX2 |
| V2LMM_88203 | CPHX |
| V2LMM_72505 | SPZ1 |
| V2LMM_63921 | OTX1 |
| V2LMM_1837 | HMX3 |
| V2LMM_58365 | ARNT |
| V2LMM_100413 | C330002I19RIK |
| V2LMM_20601 | EBF1 |
| V2LMM_86856 | HELT |
| V2LMM_44427 | PURA |
| V2LMM_36312 | ELF2 |
| V2LMM_49820 | GBX2 |
| V2LMM_35626 | NFYC |
| V2LMM_193113 | SOX14 |
| V2LMM_94805 | NR2F2 |
| V2LMM_223681 | OVOL1 |
| V2LMM_20130 | GRHL2 |
| V2LMM_66932 | KLF12 |
| V2LMM_66481 | NR2E3 |
| V2LMM_84599 | GPBP1 |
| V2LMM_39042 | TCF7 |
| V2LMM_54049 | VDR |
| V2LMM_61846 | ARX |
| V2LMM_12811 | NFYA |
| V2LMM_39033 | GTF2B |
| V2LMM_82139 | HNRPAB |
| V2LMM_8840 | PPP1R16B |
| V2LMM_62884 | SHOX2 |
| V2LMM_13844 | GSX2 |
| V2LMM_150429 | ZHX2 |
| V2LMM_21092 | DMRTA1 |
| V2LMM_36772 | ANKHD1 |
| V2LMM_153515 | POU6F1 |
| V2LMM_75704 | NR4A1 |
| V2LMM_71267 | GTF2I |
| V2LMM_3881 | CREB3L3 |
| V2LMM_16502 | FOXP2 |
| V2LMM_30875 | ZBTB48 |
| V2LMM_20855 | FOXC1 |
| V2LMM_172573 | ONECUT2 |
| V2LMM_195581 | TANC2 |
| V2LMM_212048 | RBPJ |
| V2LMM_9268 | TCF21 |
| V2LMM_11992 | NFIA |
| V2LMM_63660 | GATA5 |
| V2LMM_74122 | ZFHX3 |
| V2LMM_67224 | HOXB3 |
| V2LMM_72312 | ASB4 |
| V2LMM_204549 | AOF2 |
| V2LMM_233303 | LBA1 |
| V2LMM_74298 | LHX9 |
| V2LMM_43146 | NR4A2 |
| V2LMM_79108 | HOXA9 |
| V2LMM_18639 | ZEB1 |
| V2LMM_72786 | SLC4A10 |
| V2LMM_55316 | EGR2 |
| V2LMM_261673 | TBX10 |
| V2LMM_83123 | SOX8 |
| V2LMM_27080 | SOX11 |
| V2LMM_78560 | POU2F3 |
| V2LMM_76554 | FOXM1 |
| V2LMM_22933 | MITF |
| V2LMM_41001 | ARNTL2 |
| V2LMM_20278 | KLF15 |
| V2LMM_211741 | PRRX2 |
| V2LMM_64003 | HOXA5 |
| V2LMM_89430 | MKX |
| V2LMM_226047 | HOMEZ |
| V2LMM_55270 | NR2F2 |
| V2LMM_51703 | ASB3 |
| V2LMM_83106 | SIX4 |
| V2LMM_54745 | ASB3 |
| V2LMM_59162 | ASB15 |
| V2LMM_93309 | TANC2 |
| V2LMM_82419 | SIX1 |
| V2LMM_50340 | NR2F1 |
| V2LMM_35016 | TBR1 |
| V2LMM_43222 | DLX3 |
| V2LMM_84598 | GPBP1 |
| V2LMM_58389 | TAF5 |
| V2LMM_42883 | ESR2 |
| V2LMM_51167 | EMX2 |
| V2LMM_26703 | KRI1 |
| V2LMM_96349 | ANKIB1 |
| V2LMM_40256 | NR4A3 |
| V2LMM_27337 | ZFP110 |
| V2LMM_33336 | NFE2L1 |
| V2LMM_197153 | ANKHD1 |
| V2LMM_225761 | FOXJ1 |
| V2LMM_83480 | LSR |
| V2LMM_43261 | DMBX1 |
| V2LMM_194727 | ANKIB1 |
| V2LMM_39181 | ELF4 |
| V2LMM_34312 | E2F4 |
| V2LMM_22521 | ANKRD6 |
| V2LMM_90912 | AW146020 |
| V2LMM_57062 | GATAD2B |
| V2LMM_233535 | SOHLH1 |
| V2LMM_45265 | ERF |
| V2LMM_82719 | FKHL18 |
| V2LMM_56314 | SOX30 |
| V2LMM_54904 | SMAD3 |
| V2LMM_226780 | A030003K21RIK |
| V2LMM_194807 | TFDP2 |
| V2LMM_28937 | ELF3 |
| V2LMM_28430 | CEBPG |
| V2LMM_59103 | ZFP192 |
| V2LMM_41340 | GBX2 |
| V2LMM_43629 | EN1 |
| V2LMM_55020 | ZSCAN12 |
| V2LMM_36183 | KRI1 |
| V2LMM_13023 | NFIA |
| V2LMM_26232 | ELK1 |
| V2LMM_40531 | MRG2 |
| V2LMM_44204 | TRPC5 |
| V2LMM_36064 | STAT6 |
| V2LMM_197799 | FOXI2 |
| V2LMM_23637 | HOPX |
| V2LMM_225441 | MKX |
| V2LMM_192354 | KLF11 |
| V2LMM_91877 | E2F7 |
| V2LMM_32888 | ARNTL2 |
| V2LMM_28539 | EHF |
| V2LMM_54572 | GABPB1 |
| V2LMM_25090 | ASZ1 |
| V2LMM_226716 | TANC2 |
| V2LMM_54296 | HOXC4 |
| V2LMM_43221 | NFXL1 |
| V2LMM_34334 | ZFP354A |
| V2LMM_54213 | MEF2C |
| V2LMM_94587 | FOXN3 |
| V2LMM_46084 | ERF |
| V2LMM_189855 | HOXD8 |
| V2LMM_30266 | FOXO1 |
| V2LMM_153511 | POU6F1 |
| V2LMM_42330 | POU3F2 |
| V2LMM_45528 | PAX6 |
| V2LMM_72601 | KLF12 |
| V2LMM_77340 | FEM1C |
| V2LMM_7418 | STAT5A |
| V2LMM_162442 | TEAD1 |
| V2LMM_64850 | SIM2 |
| V2LMM_71705 | PA2G4 |
| V2LMM_122257 | HDX |
| V2LMM_174530 | GATAD2B |
| V2LMM_11542 | HDAC1 |
| V2LMM_133421 | PPP1R16B |
| V2LMM_33515 | TFDP1 |
| V2LMM_31472 | SOX11 |
| V2LMM_96120 | APOL6 |
| V2LMM_62445 | PITX2 |
| V2LMM_33008 | Smad6 |
| V2LMM_2034 | Bmi1 |
| V2LMM_32767 | Klf6 |
| V2LMM_32591 | LHX6 |
| V2LMM_172569 | ONECUT2 |
| V2LMM_25980 | AFF1 |
| V2LMM_47829 | PGR |
| V2LMM_15202 | ZEB1 |
| V2LMM_75244 | SIM1 |
| V2LMM_3247 | NFATC2 |
| V2LMM_65533 | RORC |
| V2LMM_33147 | NKX1-2 |
| V2LMM_30219 | THRB |
| V2LMM_17146 | UBTF |
| V2LMM_12422 | BARHL1 |
| V2LMM_80637 | BATF2 |
| V2LMM_204004 | C330002I19RIK |
| V2LMM_39661 | MYF5 |
| V2LMM_74588 | HNF4G |
| V2LMM_16861 | HOXB5 |
| V2LMM_62332 | NR2E3 |
| V2LMM_56311 | PSMD10 |
| V2LMM_2937 | MXD1 |
| V2LMM_44230 | DLX5 |
| V2LMM_10296 | VSX1 |
| V2LMM_22727 | TCEA2 |
| V2LMM_9662 | TGIFX1 |
| V2LMM_10940 | HOXB13 |
| V2LMM_50371 | AHR |
| V2LMM_74517 | ATF1 |
| V2LMM_16143 | BACH1 |
| V2LMM_104028 | PPP1R12B |
| V2LMM_78152 | LHX2 |
| V2LMM_134554 | E2F8 |
| V2LMM_44926 | VAX1 |
| V2LMM_82946 | NR1I2 |
| V2LMM_78883 | FOSL2 |
| V2LMM_71194 | LMX1B |
| V2LMM_37841 | ZFP263 |
| V2LMM_16518 | TWIST2 |
| V2LMM_216310 | HSF4 |
| V2LMM_6025 | MYCS |
| V2LMM_32678 | MYT1L |
| V2LMM_71097 | PRRX1 |
| V2LMM_218012 | HOXA6 |
| V2LMM_22168 | ESRRB |
| V2LMM_50036 | TRPC5 |
| V2LMM_66556 | GTF2I |
| V2LMM_64561 | ALX3 |
| V2LMM_215514 | RXRA |
| V2LMM_81583 | GCDH |
| V2LMM_79206 | ATM |
| V2LMM_202934 | ANKRD32 |
| V2LMM_67328 | ARNTL |
| V2LMM_4156 | TBX4 |
| V2LMM_72644 | NR1H3 |
| V2LMM_11508 | HES6 |
| V2LMM_36255 | ETV5 |
| V2LMM_67911 | RAX |
| V2LMM_67063 | HMX1 |
| V2LMM_6303 | NR2E1 |
| V2LMM_11527 | MSX3 |
| V2LMM_54853 | POU6F1 |
| V2LMM_2869 | NFAT5 |
| V2LMM_204235 | AOF2 |
| V2LMM_53335 | NR2F2 |
| V2LMM_67474 | MGA |
| V2LMM_58838 | BATF |
| V2LMM_48005 | PBX2 |
| V2LMM_16757 | NFKBIZ |
| V2LMM_162759 | ETS1 |
| V2LMM_71630 | HOXB3 |
| V2LMM_18746 | ZEB1 |
| V2LMM_80724 | AHCTF1 |
| V2LMM_36923 | PRRX2 |
| V2LMM_40298 | ZKSCAN3 |
| V2LMM_33294 | NFKBIE |
| V2LMM_58216 | DMRT3 |
| V2LMM_83102 | SIX4 |
| V2LMM_99408 | BTBD11 |
| V2LMM_15175 | MLX |
| V2LMM_117310 | MTF2 |
| V2LMM_49447 | EN2 |
| V2LMM_13779 | ZFP207 |
| V2LMM_128938 | KLF5 |
| V2LMM_226824 | HSF1 |
| V2LMM_65107 | HOXD4 |
| V2LMM_68412 | ARNTL |
| V2LMM_52223 | ASB3 |
| V2LMM_5501 | POLR3K |
| V2LMM_32825 | ANKRD22 |
| V2LMM_5778 | NR1D2 |
| V2LMM_27353 | ZBTB24 |
| V2LMM_92658 | SS18L1 |
| V2LMM_193584 | PBX1 |
| V2LMM_17383 | CDX4 |
| V2LMM_9964 | NFIX |
| V2LMM_60655 | GATAD2B |
| V2LMM_212472 | DBX1 |
| V2LMM_32930 | ZEB2 |
| V2LMM_59486 | SMAD3 |
| V2LMM_19590 | NR2E1 |
| V2LMM_16007 | MRG1 |
| V2LMM_76415 | ZFHX3 |
| V2LMM_15139 | MEIS1 |
| V2LMM_188087 | STAT5B |
| V2LMM_195546 | IRF6 |
| V2LMM_224682 | GPBP1 |
| V2LMM_33746 | WT1 |
| V2LMM_188544 | SUPT4H1 |
| V2LMM_218718 | PAX5 |
| V2LMM_20501 | NKX3-1 |
| V2LMM_109358 | GTF3C1 |
| V2LMM_153812 | NFIA |
| V2LMM_38234 | TFDP1 |
| V2LMM_62022 | TRPC4 |
| V2LMM_26746 | NR4A3 |
| V2LMM_34775 | ETV2 |
| V2LMM_67635 | NR0B1 |
| V2LMM_22854 | BPNT1 |
| V2LMM_25853 | ANKRD5 |
| V2LMM_67801 | NR1I3 |
| V2LMM_7016 | LHX5 |
| V2LMM_16075 | NR3C1 |
| V2LMM_173938 | ZEB2 |
| V2LMM_196876 | ANKIB1 |
| V2LMM_79613 | PAX8 |
| V2LMM_206123 | NANOG |
| V2LMM_27305 | BRF1 |
| V2LMM_167172 | ISGF3G |
| V2LMM_88779 | ZFP367 |
| V2LMM_108350 | TEAD4 |
| V2LMM_64641 | CDKN2D |
| V2LMM_159279 | IKZF3 |
| V2LMM_36580 | STAT1 |
| V2LMM_32859 | TAF7 |
| V2LMM_67344 | HOXA2 |
| V2LMM_22012 | ESRRA |
| V2LMM_31834 | ASZ1 |
| V2LMM_85443 | DMRT2 |
| V2LMM_75808 | GATA2 |
| V2LMM_81438 | THRA |
| V2LMM_59928 | OBOX3 |
| V2LMM_54957 | ZHX3 |
| V2LMM_104031 | PPP1R12B |
| V2LMM_47993 | ANKFY1 |
| V2LMM_81545 | HOXB7 |
| V2LMM_78201 | ABTB1 |
| V2LMM_30677 | ESR1 |
| V2LMM_30876 | MYCL1 |
| V2LMM_3577 | MSX3 |
| V2LMM_17167 | CREB3L3 |
| V2LMM_81141 | HOXD1 |
| V2LMM_62871 | ANKAR |
| V2LMM_92201 | TFDP2 |
| V2LMM_22072 | TAF6L |
| V2LMM_69004 | RORA |
| V2LMM_60550 | FOXF2 |
| V2LMM_43420 | VDR |
| V2LMM_69057 | FOXB1 |
| V2LMM_27291 | TCF3 |
| V2LMM_86855 | HELT |
| V2LMM_78563 | HOXB8 |
| V2LMM_111859 | GLI2 |
| V2LMM_63593 | HIF1A |
| V2LMM_56881 | ANKRD10 |
| V2LMM_26742 | TEAD1 |
| V2LMM_62312 | PAX8 |
| V2LMM_75062 | MTF2 |
| V2LMM_10700 | NRARP |
| V2LMM_29861 | NPAS2 |
| V2LMM_36256 | HOPX |
| V2LMM_53672 | FOXJ3 |
| V2LMM_188554 | RARB |
| V2LMM_188460 | EOMES |
| V2LMM_77147 | FEM1C |
| V2LMM_76338 | SNAI3 |
| V2LMM_83100 | SIX3 |
| V2LMM_196259 | SIX4 |
| V2LMM_71773 | CREB3L4 |
| V2LMM_84935 | A930001N09RIK |
| V2LMM_3446 | HSF2 |
| V2LMM_26058 | ELF2 |
| V2LMM_10570 | HOXB1 |
| V2LMM_22550 | TBX2 |
| V2LMM_75186 | TCF19 |
| V2LMM_16479 | REL |
| V2LMM_68340 | HOXA2 |
| V2LMM_47413 | ZSCAN12 |
| V2LMM_82786 | HOXC13 |
| V2LMM_56238 | PROX1 |
| V2LMM_71694 | FOXM1 |
| V2LMM_7559 | GCM1 |
| V2LMM_74227 | Klf9 |
| V2LMM_32028 | Smad5 |
| V2LMM_6901 | Cbx5 |
| V2LMM_22625 | TSHZ3 |
| V2LMM_56274 | YY1 |
| V2LMM_16891 | E2F3 |
| V2LMM_72808 | JUN |
| V2LMM_16618 | CDX2 |
| V2LMM_35782 | TEAD1 |
| V2LMM_4306 | LHX1 |
| V2LMM_92200 | TFDP2 |
| V2LMM_18851 | LASS5 |
| V2LMM_150434 | ZHX2 |
| V2LMM_156497 | GATA6 |
| V2LMM_86895 | ST18 |
| V2LMM_177663 | RORA |
| V2LMM_82787 | HOXC13 |
| V2LMM_77282 | SLC4A10 |
| V2LMM_54101 | ARNT |
| V2LMM_177662 | RORA |
| V2LMM_88647 | 6430502M16RIK |
| V2LMM_29605 | STAT1 |
| V2LMM_18745 | GTF2H1 |
| V2LMM_10822 | VSX1 |
| V2LMM_24896 | TSHZ3 |
| V2LMM_93310 | TANC2 |
| V2LMM_88651 | 6430502M16RIK |
| V2LMM_177352 | MAF |
| V2LMM_25810 | DMRTC2 |
| V2LMM_67278 | LASS6 |
| V2LMM_65504 | 4930430A15RIK |
| V2LMM_68856 | FEM1C |
| V2LMM_71630 | HOXB3 |
| V2LMM_15541 | DACH1 |
| V2LMM_173940 | ZEB2 |
| V2LMM_64594 | RXRA |
| V2LMM_196683 | HOMEZ |
| V2LMM_68185 | Ldb1 |
| V2LMM_195049 | Id1 |
| V2LMM_203104 | Rbbp8 |
| V2LMM_68372 | Brca1 |
| V2LMM_196048 | SS18L1 |
| V2LMM_204349 | NKX2-3 |
| V2LMM_50795 | DLX1 |
| V2LMM_29759 | ARNTL2 |
| V2LMM_256957 | ETV4 |
| V2LMM_261569 | ZKSCAN5 |
| V2LMM_72694 | LASS6 |
| V2LMM_218099 | IRF2 |
| V2LMM_259111 | SOX30 |
| V2LMM_11439 | ETV3 |
| V2LMM_28150 | 4930548G07RIK |
| V2LMM_72752 | 4921520G13RIK |
| V2LMM_33989 | ETV1 |
| V2LMM_162660 | ZFHX3 |
| V2LMM_76682 | ALX3 |
| V2LMM_69312 | TRPV6 |
| V2LMM_219294 | FOXJ2 |
| V2LMM_64870 | NCOR1 |
| V2LMM_5275 | NR2C1 |
| V2LMM_248905 | TBX20 |
| V2LMM_156495 | GATA6 |
| V2LMM_81731 | NR4A1 |
| V2LMM_67797 | HEY2 |
| V2LMM_84936 | A930001N09RIK |
| V2LMM_65350 | RARB |
| V2LMM_100412 | C330002I19RIK |
| V2LMM_86894 | ST18 |
| V2LMM_79339 | HOXA11 |
| V2LMM_178208 | ELF4 |
| V2LMM_83091 | SIX2 |
| V2LMM_109891 | ZFP354A |
| V2LMM_72419 | RARG |
| V2LMM_88202 | CPHX |
| V2LMM_74318 | AHCTF1 |
| V2LMM_197154 | ANKRD15 |
| V2LMM_71205 | GABPA |
| V2LMM_65377 | PITX2 |
| V2LMM_89429 | MKX |
| V2LMM_175749 | CUTL2 |
| V2LMM_69239 | ASB9 |
| V2LMM_93314 | TANC2 |
| V2LMM_77441 | DDEF1 |
| V2LMM_66404 | POU4F2 |
| V2LMM_61715 | BCOR |
| V2LMM_69801 | CREB1 |
| V2LMM_83484 | LSR |
| V2LMM_202933 | TSHZ2 |
| V2LMM_73994 | HEY1 |
| V2LMM_74506 | SP1 |
| V2LMM_108316 | CHD4 |
| V2LMM_76763 | KLF7 |
| V2LMM_189313 | CHD4 |
| V2LMM_112694 | SATB2 |
| V2LMM_178205 | ELF4 |
| V2LMM_76772 | SIM2 |
| V2LMM_70642 | ARX |
| V2LMM_51871 | AR |
| V2LMM_178206 | ELF4 |
| V2LMM_25381 | CEBPG |
| V2LMM_4254 | TBX1 |
| V2LMM_74890 | ANKFY1 |
| V2LMM_16955 | CDX2 |
| V2LMM_13423 | IRF2 |
| V2LMM_92165 | CREB3L2 |
| V2LMM_92168 | CREB3L2 |
| V2LMM_63153 | ATF3 |
| V2LMM_16955 | CDX2 |
